# Dose-Dependent Effects of Pear (*Pyrus communis* L.) Juice on Kombucha Polyphenols, Antioxidant Capacity, and Enzyme Inhibition

**DOI:** 10.3390/molecules31020371

**Published:** 2026-01-20

**Authors:** Agata Kuraj, Joanna Kolniak-Ostek

**Affiliations:** Department of Fruit, Vegetable and Plant Nutraceutical Technology, Wrocław University of Environmental and Life Sciences, 37 Chelmonskiego Street, 51-630 Wroclaw, Poland; 121401@student.upwr.edu.pl

**Keywords:** kombucha, pear juice, polyphenols, antioxidant activity, enzyme inhibition, α-glucosidase, cyclooxygenase

## Abstract

This study investigated the dose-dependent impact of pear juice supplementation on the chemical composition, phenolic profile, and biological activity of kombucha during 14 days of fermentation. Four formulations (0–75% pear juice) were evaluated for changes in (poly)phenols, organic acids, antioxidant capacity, and enzyme inhibition. UPLC-QToF-MS analysis demonstrated substantial remodeling of the phenolic profile in pear-enriched beverages, with marked increases in chlorogenic acid, arbutin, and flavonols. The total phenolic content increased proportionally with juice addition, reaching its highest level in the 75% juice formulation. Fermentation enhanced the antioxidant potential, with FRAP values more than doubling relative to the control. Pear supplementation also enhanced the inhibitory activity of key metabolic and neuroactive enzymes, including α-glucosidase, acetylcholinesterase, and butyrylcholinesterase. Principal component analysis linked phenolic enrichment to improved functional properties, highlighting the biochemical contribution of fruit-derived substrates to fermentation dynamics. Overall, the results demonstrate that pear juice acts as an effective bioactive modulator of kombucha fermentation, promoting the release, transformation, and accumulation of phenolic compounds and enhancing the antioxidant and enzyme-inhibitory potential of the beverage. These findings provide mechanistic insights into fruit-tea co-fermentation and support the development of phenolic-rich fermented beverages with improved nutritional quality and health benefits.

## 1. Introduction

Kombucha is a fermented tea beverage produced by a symbiotic culture of bacteria and yeast (SCOBY). Its chemical composition and biological activity result from complex microbial transformations that generate organic acids, vitamins, (poly)phenols, and a wide range of secondary metabolites [1,2]. Numerous studies have indicated that kombucha exhibits antioxidant, antimicrobial, and enzyme-modulating properties, which are largely attributed to the release, degradation, and biotransformation of phenolic compounds during fermentation [3,4].

The biochemical trajectory of kombucha fermentation is strongly dependent on the substrate used as the carbon and nutrient sources. Beyond traditional tea infusions, fruit juices and plant-derived matrices have recently gained attention as co-fermentation substrates, capable of enriching beverages with additional phenolic compounds, organic acids, and sensory-active metabolites. The incorporation of fruit juices, such as passion fruit, apple, grape, and pomegranate, has been shown to elevate the total phenolic content, enhance radical-scavenging activity, and diversify the metabolic profile of kombucha-like beverages [5,6,7,8]. However, emerging evidence indicates that different fruit matrices do not merely increase phenolic levels quantitatively but may drive distinct fermentation trajectories through substrate-specific phenolic structures, sugar profiles, and organic acid compositions, thereby influencing the microbial biotransformation pathways [5,7,8].

Pear (*Pyrus communis* L.) is a widely consumed fruit and a notable dietary source of chlorogenic acid, arbutin, flavonols, and proanthocyanidins, which differ structurally and functionally from the compounds predominant in other commonly studied fruits [9,10,11]. In particular, the high abundance of hydroxycinnamic acid derivatives and arbutin in pears suggests a potential for distinct bioconversion routes during fermentation, including differential ester hydrolysis, phenolic acid remodeling, and modulation of enzyme-inhibitory activity [12,13]. Unlike apples or grapes, pears have a specific balance of readily fermentable sugars, malic acid, and phenolic glycosides, which may alter microbial metabolism and phenolic transformation beyond the effects observed for other fruit-based kombucha formulations [5,7,14]. Therefore, pear juice represents not only an alternative fruit additive but also a substrate with the potential to induce qualitatively different fermentation-driven chemical and functional outcomes. Despite its phytochemical richness, the influence of pear juice as a fermentation substrate in kombucha has not been systematically studied.

Therefore, this study aimed to investigate the dose-dependent effects of pear juice supplementation on the (poly)phenol profile, antioxidant capacity, and enzyme-inhibitory properties of kombucha during fermentation, with particular emphasis on distinguishing between fruit-derived phenolics and fermentation-driven biotransformation processes. By focusing on pear-specific phenolic composition and its dynamic remodeling during fermentation, this study aimed to elucidate substrate-dependent effects that go beyond simple additive enrichment. Through the integration of chromatographic, spectrophotometric, and multivariate analyses, this study provides biochemically grounded insights into how fruit-derived substrates modulate the chemical and functional attributes of fermented tea beverages.

## 2. Results

### 2.1. Basic Chemical Composition

The initial chemical composition of the beverages reflected the proportion of pear juice in the formulations. Increasing juice content elevated the total soluble solids (TSS) and introduced slight differences in the initial pH (Table 1).

The control tea infusion (K1) exhibited 10.3 °Brix and a pH of 4.11, whereas the formulation containing 75% pear juice (K4) reached 11.25 °Brix and a pH of 4.17. These differences stem from the natural sugars and organic acids present in the pear juice.

During fermentation, the TSS values showed a transient increase on day 2 across all formulations, which is consistent with sucrose hydrolysis by yeast invertase, which liberates glucose and fructose from the disaccharide substrates. Subsequent fermentation led to a progressive decline in TSS, indicating the utilization of monosaccharides by yeasts and acetic acid bacteria for ethanol and organic acid production. By day 14, the beverages enriched with pear juice (K2-K4) exhibited TSS < 6 °Brix, which was lower than that of the control, suggesting that fruit-derived sugars accelerated microbial metabolism. Similar trends have been reported for kombucha prepared using tea infusions and fruit-based substrates [15,16].

The pH of all formulations decreased steadily throughout fermentation, reaching 3.10–3.27 on day 14. This acidification reflects the accumulation of acetic, lactic, and other organic acids and is consistent with classical kombucha fermentation pathways [17]. The substantially lower final pH observed in pear-fortified beverages supports the role of fruit-derived substrates in stimulating acidogenesis, which is a phenomenon also reported for kombucha produced using fruit juices [7,16]. Importantly, the final pH values were well below 3.5, which is associated with enhanced microbial stability and safety [4,18].

Sucrose, the main carbohydrate source, was rapidly hydrolyzed in all formulations and was undetectable by day 14 (Table 1). In the 25% pear juice variant (K2), sucrose was depleted as early as day 4, whereas traces persisted until day 8 in the other formulations. This rapid decline confirms effective sucrose inversion in pear-enriched beverages, which is consistent with previous observations indicating accelerated sugar utilization in kombucha supplemented with fruit substrates [19].

The dynamics of the monosaccharide release differed among the variants. The formulations containing pear juice (K2–K4) exhibited higher initial glucose and fructose levels than the control, reflecting the intrinsic sugar composition of pears. In K3 and K4, both monosaccharides continuously declined during fermentation, indicating an efficient microbial assimilation. Conversely, the control beverage (K1) showed an increase in glucose and fructose levels by day 14, likely due to ongoing sucrose hydrolysis which exceeded the rate of microbial uptake. In K2, glucose increased, whereas fructose decreased, suggesting potential preferential fructose utilization by the microbial consortium, which is a phenomenon previously linked to the differential substrate affinity of *Saccharomyces* spp. and acetic acid bacteria [12,20].

Fermentation in all variants was accompanied by a gradual accumulation of organic acids. Acetic acid was the dominant acid, reaching 0.42–0.48 mg/100 mL by day 14, confirming the activity of acetic acid bacteria as key drivers of kombucha metabolism. These values fall within the lower range of concentrations reported in the literature, which vary widely depending on substrate composition and microbial ecology [9,14,21]. Lactic acid was present at lower concentrations (~0.05 mg/100 mL), indicating the metabolic activity of lactic acid bacteria within the SCOBY consortium [17].

Malic acid, naturally abundant in pears, was absent in the control (K1) but was consistently detected in pear-fortified formulations (K2–K4) from the start of fermentation. Its persistence throughout the process confirms the direct contribution of fruit-derived metabolites to the acid profile of the beverage, which is consistent with previous studies showing that malic acid can serve as both a microbial substrate and a contributor to flavor and acidity in fruit-enriched kombucha [7,22].

The basic chemical composition of the beverages was influenced by both microbial activity and pear juice supplementation. Enhanced sugar turnover and the presence of fruit-derived organic acids underscore the significance of fruit–tea co-fermentation in modulating fermentation kinetics, acidogenesis, and nutritional characteristics of the final beverage.

### 2.2. Microbial Contributions and Functional Implications

The biochemical transformations observed during fermentation are the result of the coordinated metabolic activity of yeasts, acetic acid bacteria (AAB), and lactic acid bacteria (LAB) within the SCOBY consortium. Yeasts initiate this process by hydrolyzing sucrose into glucose and fructose and converting these monosaccharides into ethanol, carbon dioxide, and intermediate metabolites. Ethanol subsequently serves as a substrate for AAB, which oxidizes it into acetic acid and acetaldehyde, thereby contributing to the characteristic acidity and flavor profile of kombucha [23]. The increasing acetic acid concentrations observed across the formulations are consistent with this classical metabolic route and suggest an active role of AAB in sugar-derived acidogenesis.

Although LAB generates lactic acid at markedly lower concentrations than AAB-produced acetic acid, their metabolic contribution extends beyond acidification. LAB are known to produce a variety of bioactive molecules, including peptides, γ-aminobutyric acid (GABA), and exopolysaccharides, which can enhance the functional properties and structural stability of fermented beverages [24,25]. The presence of readily fermentable sugars and organic acids in pear juice may have favored LAB activity, thereby enriching cross-feeding interactions within the SCOBY and contributing to the metabolic complexity of the pear-enriched formulations.

The detection of malic acid in pear juice-containing variants provides additional insights into the contributions of microorganisms. Malic acid, naturally abundant in pears, may undergo partial malolactic conversion to lactic acid, a process well documented in wine fermentation and facilitated by certain LAB species [26]. Although direct evidence of malolactic fermentation was not obtained in this study, the persistence of malic acid alongside low but detectable lactic acid concentrations is compatible with limited LAB-mediated conversion, supporting the presence of metabolically active LAB capable of modulating both acidity and flavor. The simultaneous presence of acetic, lactic, and malic acids in pear-enriched beverages suggests a diverse acid profile, frequently associated with improved sensory properties. In contrast, excessive acetic acid accumulation may impart an undesirable vinegar-like flavor [27].

Importantly, the interpretation of microbial contributions in this study was based on established kombucha fermentation pathways and indirect metabolic markers rather than direct microbiological analyses. The SCOBY composition was not characterized at the species or strain level, and microbial dynamics were inferred from changes in sugar, organic acid, and phenolic compound levels. Therefore, the proposed links between pear juice supplementation, microbial activity, and biochemical outcomes should be regarded as hypothesis-generating rather than causative links. Future studies integrating microbial profiling techniques, such as high-throughput sequencing or targeted cultivation approaches, are necessary to directly elucidate pear-specific microbial–substrate interactions during kombucha fermentation.

Collectively, these findings indicate that pear juice supplementation modulates not only the chemical composition but also the inferred metabolic activity of kombucha. Fruit-derived substrates enhance the availability of fermentable carbohydrates and organic acids, potentially stimulating distinct microbial pathways and diversifying metabolic outputs. Such interactions may contribute to the desirable sensory attributes, reduced residual sugar content, and potentially enhanced health-related properties of fruit-tea co-fermented beverages.

### 2.3. Polyphenolic Compounds

UPLC-QToF analysis demonstrated that the polyphenolic profile of the kombucha beverages was strongly dependent on the proportion of pear juice incorporated into the formulations (Supplementary Table S1). Importantly, the observed changes were not limited to simple quantitative enrichment but reflected qualitative remodeling of the phenolic matrix as a function of substrate composition. The control variant (K1), consisting solely of green tea, contained characteristic *Camellia sinensis*-derived phenolics, such as catechins, epigallocatechin gallate, theaflavins, and quercetin glycosides, in agreement with the published profiles of tea-based beverages [28]. The addition of pear juice (K2-K4) introduced compounds typical of pear phytochemistry, particularly hydroxycinnamic acid derivatives (e.g., caffeoylquinic acids and caffeoylhexosides) and procyanidins. Notably, several pear-associated metabolites, including caffeoyl-N-tryptophan and isorhamnetin derivatives, exhibited formulation-dependent occurrence and accumulation, indicating selective microbial accessibility rather than uniform transfer from the juice matrix. Several compounds, including caffeoyl-N-tryptophan and isorhamnetin derivatives, were predominantly or exclusively detected in the 75% pear juice formulation (K4). These results are consistent with earlier characterizations of pears as rich sources of caffeic acid derivatives and proanthocyanidins [9,10,11].

Fermentation markedly altered the concentrations of individual phenolic compounds and their respective subclasses (Table 2; Supplementary Tables S2–S5). In K1-K3, the total polyphenol content (TPC) increased over the 14-day period, suggesting the enhanced release of bound phenolics and microbial biotransformation of conjugated structures into more extractable forms. This trend indicates that moderate pear supplementation promotes fermentation-driven phenolic liberation, rather than merely increasing the initial phenolic load. Similar increases in phenolic availability during kombucha fermentation have been previously reported [2,7]. In contrast, the TPC in the high-juice formulation (K4) remained relatively stable and slightly decreased. This divergent trajectory suggests that high concentrations of pear-derived phenolics may follow alternative degradation or transformation pathways, potentially involving polymer breakdown or microbial utilization as secondary carbon sources. This behavior likely reflects the structural characteristics of pear-derived phenolics, including their lower susceptibility to enzymatic hydrolysis and the potential degradation of fruit procyanidins during extended fermentation.

Hydroxycinnamic acids exhibited distinct formulation-dependent changes. Their total levels decreased in K1 and K4 but increased in K2 and K3, with a notable increase in 3-p-coumaroylquinic acid levels. The selective enrichment of this compound in intermediate pear–tea systems suggests a substrate-specific bioconversion pathway that was not observed in tea-only or pear-dominant matrices. This enrichment likely results from substrate-specific microbial transformations, as both tea and pear juices supply precursors for the synthesis and release of hydroxycinnamate derivatives. These bidirectional changes, the degradation of some chlorogenic derivatives versus the formation of newly detectable compounds, reflect the dual nature of the fermentation effects described for kombucha and other fermented substrates [13]. Metabolomic studies have demonstrated that hydroxycinnamates undergo extensive deconjugation, ester hydrolysis, and secondary derivative formation during kombucha fermentation [12].

Flavan-3-ols and proanthocyanidins increased most prominently in the tea-dominated beverages, rising by 22.1% in K1 and 14.1% in K2, whereas only modest increases occurred in K3 (6.4%) and K4 (2.9%) in the other beverages. This gradient highlights that pear juice does not simply dilute tea-derived catechins but actively reshapes their fermentation fate by modifying the precursor availability and microbial metabolic priorities. This pattern underscores green tea as the primary source of catechins and procyanidins, whereas pear juice contributes additional phenolics without supporting the same degree of microbial release or structural modification as green tea does. The attenuated rise in K4 likely reflects both the reduced proportion of tea-derived precursors and the potential microbial depolymerization or conversion of fruit-origin procyanidins, which is a phenomenon that has been previously observed in fermented fruit matrices [14].

Flavonol levels, mainly quercetin glycosides, remained relatively stable across formulations, indicating limited susceptibility to SCOBY-mediated hydrolysis. This stability contrasts with the dynamic behavior of phenolic acids and supports the concept of compound-class-specific fermentation trajectories. In contrast, phenolic acids and their derivatives responded more dynamically to fermentation, displaying either enrichment or depletion depending, on the substrate composition and microbial enzymatic activity. These variable patterns align with previous evidence that sugar concentration and carbon availability modulate the metabolic pathways involved in phenolic transformation [19].

The mechanistic basis for these transformations can be linked to specific microbial processes previously documented in kombucha systems. Yeasts and bacteria catalyze deglycosylation, ester hydrolysis, reductive and oxidative conversions, and the formation of secondary phenolic derivatives [29]. However, in the present study, the direction and magnitude of these transformations were strongly influenced by pear-specific phenolic structures, indicating that the fruit matrix acts as a metabolic modulator rather than a passive additive. More advanced metabolomic investigations have confirmed that kombucha fermentation involves a complex cascade of bioconversion reactions affecting simple phenolic acids, flavan-3-ols, and high-molecular-weight proanthocyanidins [30]. Furthermore, studies on fruit-enriched kombucha beverages emphasize that the interaction between tea-derived and fruit-derived phenolics results in matrix-specific transformation pathways that are not observed in either substrate alone [5].

Kombucha fermentation selectively modulates the phenolic composition through a combination of release, degradation, structural transformation, and substrate-specific microbial bio-conversion. Collectively, these results indicate that pear juice induces distinct phenolic remodeling patterns rather than generic enrichment effects, thereby supporting a substrate-dependent fermentation model. While the total phenolic content increased in tea-based and moderately fruit-fortified variants, the magnitude and trajectory of the changes were strongly influenced by the relative proportion of tea and pear juice, highlighting the substrate dependence of the polyphenolic biotransformation pathways.

Importantly, the observed polyphenolic changes cannot be interpreted as a simple additive transfer of pear-derived compounds to the kombucha matrix. Pear juice supplementation introduced a phenolic profile dominated by hydroxycinnamic acids, arbutin-related structures, and specific flavonol derivatives characteristic of *Pyrus communis*, which differed substantially from the phenolic spectra reported for apple-, grape-, or pomegranate-based kombucha systems [5,7,9,10,11]. During fermentation, these pear-specific phenolics exhibited distinct transformation trajectories, including the selective stability of flavonols, limited net increases in proanthocyanidins, and bidirectional remodeling of chlorogenic acid derivatives, suggesting substrate-dependent bioconversion rather than uniform phenolic release. In particular, the enrichment of specific caffeoylquinic and p-coumaroylquinic acids in intermediate juice formulations (K2–K3), in contrast to their partial depletion in the highest juice variant (K4), indicates that the fate of phenolics is modulated by the relative balance between tea- and pear-derived precursors, sugar availability, and fermentation progression. Similar matrix-specific transformation patterns have been reported in metabolomic studies of kombucha fermentation, where hydroxycinnamates undergo deconjugation, ester hydrolysis, and secondary derivative formation, depending on the substrate composition [12,13]. Therefore, the present findings suggest that pear juice acts not only as a source of additional phenolics but also as a modulator of fermentation-driven polyphenolic remodeling, shaping compound-specific stability and transformation pathways that cannot be directly extrapolated from other fruit–kombucha systems [5].

### 2.4. Antioxidant Capacity

Fermentation significantly influenced the antioxidant capacity of the beverages, with clear differences observed among the formulations (Table 3). On day 0, samples containing pear juice (K2-K4) exhibited higher ABTS, FRAP, and DPPH values than the tea-only control (K1), reflecting the contribution of fruit-derived phenolics with strong radical-scavenging properties. During fermentation, the antioxidant activity showed fluctuations but ultimately increased by day 14 in all variants, with the most pronounced increase observed in juice-enriched formulations.

The 75% pear juice beverage (K4) showed the highest final antioxidant capacity (3855.47, 2669.62, and 1970.92 μmol Trolox/100 mL in ABTS, FRAP, and DPPH assays, respectively), whereas K1 remained significantly lower (2121.28, 639.29, and 860.92 μmol Trolox/100 mL). These results confirm the synergistic contribution of green tea catechins and pear-derived (poly)phenols, and are consistent with previous reports on kombucha and lactic acid-fermented fruit matrices [2,21].

The increase in antioxidant capacity during fermentation can be attributed to several mechanisms: (1) enzymatic release of bound phenolics from tea and pear tissues, (2) microbial biotransformation of complex phenolics into lower-molecular-weight compounds with higher reducing power, and (3) formation of fermentation-derived metabolites, such as organic acids, which may synergistically enhance antioxidant responses.

These mechanisms align with previously noted increases in antioxidant activity during the fermentation of pear juice and kombucha-like beverages [7,14], highlighting the role of pear juice in enhancing both the initial antioxidant potential and fermentation-driven amplification.

### 2.5. Enzyme Inhibitory Activities

Fermentation significantly enhanced the enzyme-inhibitory activities of all formulations (Table 3). On day 14, α-amylase and α-glucosidase inhibition increased in all variants, with K4 showing the strongest effect (35.89% and 40.58%, respectively), followed by K3 (30.41% and 30.99%), K2 (25.67% and 29.22%), and K1 (22.15% and 25.01%), respectively. Although enzyme inhibition was assessed at a single concentration and did not allow the determination of IC_50_ values, the applied approach provides a comparative, screening-level insight into the relative bioactivity changes induced by fermentation and substrate composition. These results indicate that fermentation promotes the formation or release of phenolic inhibitors of carbohydrate-hydrolyzing enzymes, particularly chlorogenic, gallic, and caffeic acid derivatives, which have been consistently associated with antidiabetic functionality in fermented fruit beverages [31,32]. The dose-dependent strengthening of inhibition across K1–K4 further suggests that pear juice supplementation modulates not only the absolute phenolic content but also the qualitative profile of the low-molecular-weight inhibitors generated during fermentation.

Pear-enriched beverages also exhibited enhanced inhibition of AChE and BuChE, with the strongest effects recorded in K4 (13.12% for AChE and 73.39% for BuChE) and the weakest in K1 (8.08% and 44.85%, respectively). This pattern cannot be explained by the simple additive effects of pear phenolics alone, as fermentation stage-dependent increases were also observed within individual formulations. This trend may be attributed to the presence of pear phenolics, such as quercetin, isorhamnetin derivatives, and chlorogenic acid, and microbial metabolites generated during fermentation. These compounds have been associated with neuroprotective potential in fruit-based and fermented food products [33,34].

The stronger response of BuChE compared to AChE may reflect the differential sensitivity of these enzymes to phenolic acids and flavonols, as previously reported for plant-derived inhibitors.

Similarly, COX-1 and COX-2 inhibition increased significantly during the fermentation process. The highest activities were observed in K4 (94.12% and 95.34%), and the lowest were observed in K1 (80.39% and 83.70%), respectively. These findings align with reports indicating that polyphenol-rich kombucha metabolites exert anti-inflammatory effects, partly mediated by the modulation of downstream signaling pathways [35]. The progressive increase in COX inhibition along the pear juice gradient suggests that fruit-specific hydroxycinnamic acids and their fermentation-derived metabolites may play a complementary role to tea-derived flavonoids in shaping the anti-inflammatory potential. Overall, these results demonstrate that pear juice fortification substantially enhances the biological activity of kombucha, particularly in the pear-dominant formulation (K4). Importantly, the observed effects reflect substrate-dependent fermentation outcomes rather than a direct linear transfer of fruit bioactivity, indicating that microbial biotransformation is a key driver of the enzyme inhibition patterns. The enhancement across antioxidant, antidiabetic, anticholinergic, and anti-inflammatory assays highlights the synergistic interplay between fruit-derived substrates and microbial metabolism in shaping the functional potential of kombucha-type beverages.

The combined analysis of phenolic composition, antioxidant capacity, and enzyme-inhibitory activities demonstrated that the functional properties of kombucha beverages are closely linked to the fermentation-driven transformations of both tea- and pear-derived phenolics. The observed increase in antioxidant activity paralleled the increase in total polyphenol content in most formulations, supporting the notion that microbial hydrolysis and bioconversion enhance the availability of phenolic compounds with strong reducing power [2,7,14]. Variations in the behavior of individual phenolic subclasses, such as the formation of specific hydroxycinnamic acid derivatives in K2 and K3 or the limited increase in procyanidins in K4, highlight the substrate-dependent nature of microbial metabolism and align with earlier evidence that sugar concentration and substrate composition modulate phenolic transformations [13,19]. These structural changes are reflected in the biological activity of the beverages, where the enhanced inhibition of α-amylase and α-glucosidase corresponds to the increased abundance of low-molecular-weight phenolic acids, which are well documented for their capacity to modulate carbohydrate-digesting enzymes [31,32]. Similarly, enhanced AChE and BuChE inhibition in pear-enriched variants may be attributed to the presence of quercetin, chlorogenic acid, and their derivatives, which exhibit neuroprotective potential in fruit-based and fermented products [33,34]. The strong inhibition of COX-1 and COX-2 observed, particularly in K4, further supports the contribution of phenolic compounds and fermentation-derived metabolites to anti-inflammatory responses [35]. Taken together, these findings support a mechanistic framework in which pear juice acts as a modulatory fermentation substrate, shaping enzyme inhibition selective phenolic remodeling rather than through simple enrichment.

It should be emphasized that the observed improvements in polyphenolic composition, antioxidant capacity, and enzyme inhibitory activity cannot be solely attributed to the intrinsic properties of pear juice. Instead, the results reflect fermentation-driven transformations occurring within a complex tea–fruit matrix. Although a non-fermented pear juice control was not included in the experimental design, the dynamic changes observed over the fermentation period, particularly the temporal remodeling of individual phenolic subclasses, organic acid profiles, and biological activities, clearly indicated an active contribution of microbial metabolism rather than a simple additive effect of fruit supplementation. Notably, several phenolic compounds and derivatives detected in pear-enriched kombucha variants were either absent or present at significantly lower levels on day 0 but emerged or increased during fermentation, supporting the role of SCOBY-mediated biotransformation processes. Similar fermentation-dependent enhancement of bioactivity, exceeding the effect of raw fruit substrates alone, has been reported for other fruit–tea kombucha systems [5,6,7,8]. Therefore, the present study was designed to elucidate the substrate-dependent fermentation effects, rather than comparing kombucha with non-fermented pear juice. Future studies incorporating unfermented fruit juice controls and targeted microbial profiling would further clarify the distinction between additive and synergistic effects and allow a quantitative assessment of the fermentation-induced bioactivation of pear-derived phenolics.

### 2.6. Principal Component and Correlation Analyses

**Figure 1:**
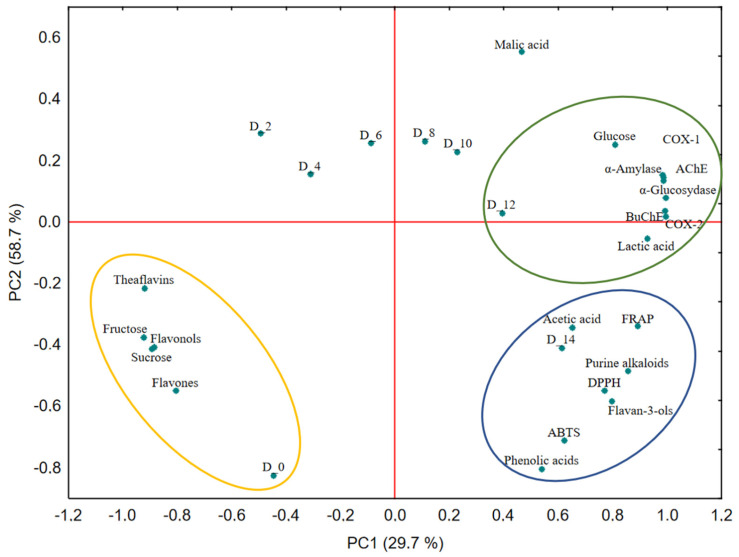
Principal component analysis (PCA) biplot of the physicochemical parameters, phenolic compounds, and biological activities of kombucha beverages during 14-day fermentation (D_0–D_14). Ellipses represent the 95% confidence regions for the main fermentation stages.

Principal component analysis (PCA) was performed to integrate the changes in chemical composition and biological activity during the fermentation period (Figure 1). The first two principal components (PC1 = 29.7%; PC2 = 58.7%) explained 88.4% of the total variance, indicating a robust multivariate model. The temporal distribution of the samples revealed three distinct fermentation phases.

During the early stage (D_0–D_4), samples clustered in association with high sucrose and fructose levels and elevated concentrations of tea-derived flavonoids (theaflavins, flavonols, and flavones). This pattern reflects a system dominated by raw substrate components, with limited microbial transformations.

In the intermediate stage (D_6–D_10), samples shifted toward associations with glucose, malic acid, and enhanced biological activities, including α-amylase, α-glucosidase, acetylcholinesterase, BuChE, COX-1, and COX-2 inhibition. This phase represents the onset of intensified biochemical activity, marked by active microbial metabolism, accelerated sugar conversion, and the emergence of bioactive phenolic derivatives that contribute to antidiabetic, neuroprotective, and anti-inflammatory properties.

The final stage (D_12–D_14) was clustered with lactic and acetic acids, flavan-3-ols, purine alkaloids, and diverse phenolic acids, coinciding with the peak antioxidant capacity in ABTS, DPPH, and FRAP assays. This indicates a mature fermentation state in which phenolic release, biotransformation, and organic acid accumulation collectively enhance functional potential.

**Figure 2:**
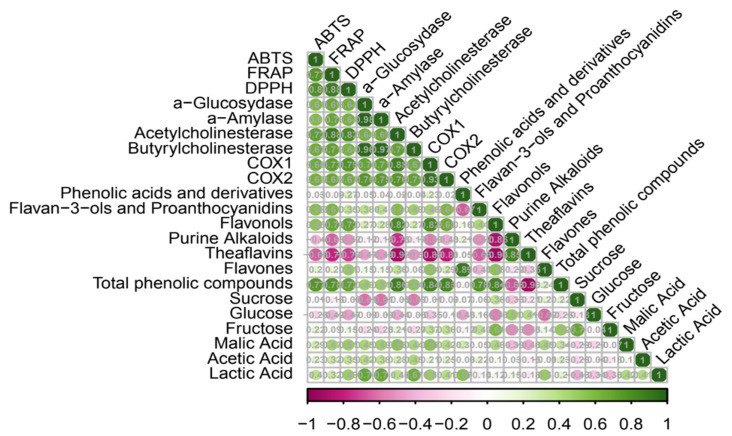
Pearson correlation heatmap showing the relationships between the physicochemical variables, phenolic compounds, and biological activities of kombucha beverages.

Correlation analysis (Figure 2) supported the trends observed in PCA-derived. Antioxidant capacity demonstrated strong positive correlations with total phenolic content (r ≈ 0.7), flavan-3-ols (r ≈ 0.6), and flavonols (r ≈ 0.7), confirming their central role in the redox activity. Enzyme inhibition (α-amylase and α-glucosidase) correlated strongly with total phenolics and flavonols (r > 0.80), consistent with the established contribution of these compounds to antidiabetic mechanisms [31,32]. AChE and BuChE inhibition showed positive associations with flavan-3-ols, flavonols, and organic acids and aligned closely with COX-1/COX-2 activity, suggesting overlapping neuroprotective and anti-inflammatory pathways, which is consistent with previous findings on phenolic-rich fermented beverages [33,34,35].

Negative correlations between sucrose/fructose and all biological activities underscore the importance of sugar depletion as a trigger for the accumulation of functional metabolites, including transformed phenolics and fermentation-derived organic acids. Together, PCA and correlation analyses indicated that fermentation drives a systematic shift from raw substrate–dominated profiles to metabolite-enriched, functionally potent beverages. Phenolic compounds, particularly flavan-3-ols, flavonols, and phenolic acids, along with acetic and lactic acids, emerged as key markers underpinning the antioxidant, antidiabetic, anti-inflammatory, and neuroprotective properties of the pear-fortified kombucha.

## 3. Materials and Methods

### 3.1. Preparation of Kombucha Beverages

Green tea (*Camellia sinensis* L. var. Gunpowder) was obtained from a commercial supplier (PROGRESSIVE Sp. z o.o, Wrocław, Poland). Fresh pears (*Pyrus communis* L., cv. ‘Conference’) were sourced from a local orchard (Lower Silesia, Poland). All analytical-grade reagents and chromatographic standards were purchased from Sigma-Aldrich (St. Louis, MO, USA) and Merck (Darmstadt, Germany).

A laboratory-maintained symbiotic culture of bacteria and yeast (SCOBY) was used for fermentation. The same SCOBY stock was used for all the treatments to ensure a uniform microbial composition. The consortium predominantly consisted of *Saccharomyces*, *Schizosaccharomyces*, *Acetobacter*, and *Lactobacillus* spp., which are commonly associated with kombucha fermentation. No pathogenic or biosafety level 2 microorganisms were observed. All procedures followed the institutional biosafety guidelines.

Sweetened tea infusions were prepared by steeping 100 g of green tea leaves in 1 L of boiling water for 20 min, followed by filtration through a Whatman paper. A 5 L bulk infusion was prepared, supplemented with 10% (*w*/*v*) sucrose, and cooled to room temperature.

Pear juice was extracted using a laboratory press, pasteurized at 90 °C for 10 min, cooled, and stored until use. A single 3 L batch was prepared to ensure uniformity. Juice was incorporated into the cooled tea infusions prior to inoculation to yield four formulations: K1 (control): 100% tea; K2: 75% tea + 25% pear juice; K3: 50% tea + 50% pear juice; and K4: 25% tea + 75% pear juice.

Each 2 L formulation was inoculated with 10% (*v*/*v*) pre-fermented kombucha as a starter culture. Fermentation was carried out under static conditions at 20 °C in sterile 2 L glass jars covered with breathable, sterile gauze. All samples were protected from light. Aliquots were collected on day 0 (D_0) and every two days thereafter (D_2–D_14) for chemical and biological analyses.

### 3.2. Physicochemical Analyses

The pH of the samples was measured using a DL-21 automatic titrator (Mettler-Toledo, Schwerzenbach, Switzerland). Total soluble solids (TSS) were determined using an Atago PR-101 digital refractometer (Atago Co., Ltd., Tokyo, Japan) and expressed in °Brix units. All measurements were performed in triplicate using three independent fermentation experiments for each measurement.

### 3.3. Determination of Organic Acids and Sugars

Sugar extraction was performed as described by Kolniak-Ostek [9]. Chromatographic separation was performed using a Merck-Hitachi L-7455 HPLC system (Merck KgaA, Darmstadt, Germany), equipped with an evaporative light-scattering detector (ELSD; PL-ELS 1000, Merck), L-7100 quaternary pump (Merck), and L-7200 autosampler (Merck). Sugars were resolved on a Prevail Carbohydrate ES column (250 × 4.6 mm, 5 µm; Alltech, Deerfield, IL, USA). Calibration curves (R^2^ = 0.9999) were generated for glucose, fructose, sucrose, and sorbitol. The results were expressed as mg/100 mL.

Organic acids were determined using a Dionex Ultimate 3000 HPLC system (Thermo Fisher Scientific, Sunnyvale, CA, USA) equipped with an LPG-3400A pump (Thermo Fisher Scientific) and TCC-3000SD (Thermo Fisher Scientific) column oven, as described by Kolniak-Ostek [9]. Separation was achieved on an Aminex HPH-87H column (300 × 7.8 mm; Bio-Rad, Hercules, CA, USA) with an IG Cation H guard column (30 × 4.6 mm). The mobile phase was 0.001 N H_2_SO_4_ delivered isocratically at 0.6 mL/min. Detection was performed at 210 nm. All analyses were conducted in triplicate using three independent biological replicates.

### 3.4. Determination of Polyphenolic Compounds

Polyphenolic profiles were determined using an ACQUITY UPLC system (Waters, Manchester, UK) equipped with a photodiode-array detector (PDA) and a G2 Q-ToF mass spectrometer with an electrospray ionization (ESI) source operating in both positive and negative ion modes [10]. Separation was performed on an ACQUITY UPLC BEH C18 column (100 × 2.1 mm, 1.7 µm; Waters) at 40 °C. The mobile phases were (A) 0.1% formic acid in water and (B) acetonitrile, with a gradient from 5% to 40% B over 30 min at 0.35 mL/min. Mass spectra were recorded in the *m*/*z* 100–1500 range. The identification was based on retention times, UV-Vis spectra, and MS/MS fragmentation patterns, and confirmed using authentic standards and literature data [11]. The results were expressed as mg/100 mL.

### 3.5. Determination of Biological Activities

Antioxidant activity was quantified using the ABTS, DPPH, and FRAP assays according to Kolniak-Ostek et al. [36], and the results were expressed as µmol Trolox equivalents per 100 mL.

The anti-inflammatory potential was assessed by measuring the inhibition of cyclooxygenase isoenzymes (COX-1 and COX-2) following the method described by Pawluś and Kolniak-Ostek [19]. Antidiabetic activity was evaluated using α-amylase and α-glucosidase inhibition assays, as described by Podsędek et al. [37]. Cholinesterase inhibition (AChE and BuChE) was determined using the colorimetric method described by Rhee et al. [38]. All assays were performed in triplicate using three independent fermentation replicates.

Enzyme inhibition assays were conducted at a single extract concentration (1 mg/mL) and were intended as comparative screening tests rather than for determining pharmacological potency. This experimental design was selected to allow a direct comparison of the relative changes in the inhibitory potential induced by fermentation and pear juice supplementation within a complex food matrix. Determination of IC_50_ values was beyond the scope of this study, as the primary objective was to evaluate the fermentation-driven modulation of bioactivity rather than the dose–response relationships of individual compounds.

### 3.6. Statistical Analysis

Data are presented as mean ± standard deviation (SD). Differences between treatments were evaluated using one-way ANOVA followed by Duncan’s post hoc test (*p* < 0.05) using Statistica 13.3 (StatSoft, Tulsa, OK, USA). Principal component analysis (PCA) was performed using R version 4.3.1 and the Factoextra 1.0.7 package.

## 4. Conclusions

This study demonstrates that fortifying kombucha with pear (*Pyrus communis* L.) juice significantly alters its chemical composition and enhances its functional properties. Increasing the proportion of pear juice increased the total polyphenol content, diversified the phenolic profile, and strengthened the antioxidant, antidiabetic, anti-inflammatory, and neuroprotective activities. The most pronounced effects were observed in the 75% juice formulation (K4), indicating that pear juice provides additional phenolic substrates and readily fermentable sugars that intensify the microbial metabolism and drive fermentation-related biotransformation. These findings highlight the importance of fruit–tea co-fermentation as a strategy for modulating the phenolic composition and shape the biological potential of kombucha beverages through substrate-dependent microbial activity. Future studies should investigate the influence of different fruit matrices, tea types, and microbial communities on phenolic transformations and functional properties, and assess their potential in the context of targeted nutritional applications.

## Figures and Tables

**Table 1:** pH, total soluble solids (°Brix), reducing sugars (mg/100 mL) and organic acids (mg/100 mL) in the tested kombucha beverages during fermentation.

				Reducing Sugars			Organic Acids		
		pH	Total Soluble Solids	Fructose	Glucose	Sucrose	Acetic Acid	Malic Acid	Lactic Acid
K1	Day 0	4.11 ± 0.00 ^b^	10.30 ± 0.28 ^de^	6.00 ± 0.03 ^k^	4.99 ± 0.02 ^j^	239.97 ± 3.56 ^f^	0.41 ± 0.00 ^b^	ND	ND
	Day 2	4.02 ± 0.00 ^c^	11.90 ± 0.00 ^a^	7.70 ± 0.09 ^k^	12.21 ± 0.08 ^i^	197.96 ± 2.11 ^g^	0.37 ± 0.00 ^c^	ND	ND
	Day 4	3.57 ± 0.01 ^ef^	10.70 ± 0.00 ^d^	9.68 ± 0.10 ^k^	20.80 ± 0.09 ^g^	51.94 ± 1.21 ^j^	0.18 ± 0.01 ^fg^	0.15 ± 0.00 ^fg^	ND
	Day 6	3.39 ± 0.00 ^g^	10.10 ± 0.00 ^e^	3.31 ± 0.15 ^kl^	87.96 ± 1.03 ^c^	61.33 ± 1.12 ^i^	0.31 ± 0.01 ^de^	0.28 ± 0.01 ^cd^	ND
	Day 8	3.31 ± 0.00 ^h^	7.70 ± 0.00 ^h^	23.28 ± 0.13 ^i^	170.53 ± 2.56 ^a^	18.50 ± 0.13 ^l^	0.35 ± 0.02 ^c^	0.33 ± 0.01 ^b^	ND
	Day 10	3.22 ± 0.00 ^i^	7.20 ± 0.00 ^hi^	17.55 ± 0.11 ^ij^	157.86 ± 3.09 ^b^	ND	0.26 ± 0.01 ^e^	0.27 ± 001 ^cd^	ND
	Day 12	3.17 ± 0.01 ^i^	6.95 ± 0.07 ^i^	14.54 ± 0.15 ^j^	164.13 ± 2.66 ^ab^	ND	0.28 ± 0.00 ^e^	ND	ND
	Day 14	3.10 ± 0.00 ^j^	7.45 ± 0.07 ^hi^	13.1 ± 0.11 ^j^	167.93 ± 3.05 ^a^	ND	0.48 ± 0.02 ^a^	ND	0.03 ± 0.00 ^bc^
K2	Day 0	4.12 ± 0.00 ^b^	10.90 ± 0.00 ^cd^	71.10 ± 0.36 ^e^	16.60 ± 0.41 ^h^	266.99 ± 1.25 ^e^	0.34 ± 0.02 ^cd^	0.14 ± 0.00 ^fg^	ND
	Day 2	4.09 ± 0.00 ^bc^	11.50 ± 0.00 ^b^	72.02 ± 0.85 ^e^	24.17 ± 0.22 ^g^	97.19 ± 1.36 ^h^	0.35 ± 0.01 ^c^	0.16 ± 0.00 ^f^	ND
	Day 4	3.58 ± 0.01 ^e^	10.80 ± 0.00 ^d^	46.50 ± 0.69 ^g^	24.42 ± 0.16 ^g^	53.76 ± 1.20 ^j^	0.22 ± 0.01 ^f^	0.20 ± 0.01 ^ef^	ND
	Day 6	3.48 ± 0.00 ^f^	8.25 ± 0.00 ^g^	47.05 ± 0.48 ^g^	40.96 ± 0.32 ^ef^	ND	0.38 ± 0.02 ^bc^	0.32 ± 0.01 ^bc^	ND
	Day 8	3.36 ± 0.00 ^gh^	7.20 ± 0.00 ^hi^	48.06 ± 0.32 ^fg^	44.96 ± 0.28 ^e^	ND	0.35 ± 0.01 ^c^	0.30 ± 0.02 ^c^	ND
	Day 10	3.28 ± 0.00 ^hi^	6.90 ± 0.00 ^i^	56.54 ± 0.56 ^f^	34.80 ± 0.26 ^f^	ND	0.33 ± 0.00 ^d^	0.22 ± 0.01 ^e^	ND
	Day 12	3.19 ± 0.00 ^i^	6.35 ± 0.07 ^ij^	70.12 ± 1.06 ^e^	34.19 ± 0.15 ^f^	ND	0.36 ± 0.01 ^c^	0.22 ± 0.01 ^e^	0.02 ± 0.00 ^cd^
	Day 14	3.12 ± 0.00 ^j^	5.55 ± 0.07 ^k^	58.64 ± 1.22 ^f^	18.45 ± 0.11 ^h^	ND	0.34 ± 0.01 ^cd^	0.22 ± 0.01 ^e^	0.03 ± 0.01 ^bc^
K3	Day 0	4.12 ± 0.00 ^b^	10.95 ± 0.07 ^cd^	179.6 ± 3.12 ^c^	22.44 ± 0.32 ^g^	495.34 ± 2.56 ^a^	0.31 ± 0.01 ^d^	0.17 ± 0.01 ^f^	-
	Day 2	4.07 ± 0.01 ^bc^	11.80 ± 0.14 ^a^	80.37 ± 1.84 ^e^	11.59 ± 0.22 ^1^	469.49 ± 3.15 ^b^	0.21 ± 0.01 ^f^	0.12 ± 0.00 ^g^	-
	Day 4	3.63 ± 0.00 ^de^	8.60 ± 0.00 ^g^	76.76 ± 2.16 ^e^	15.76 ± 0.19 ^h^	245.41 ± 2.69 ^f^	0.32 ± 0.01 ^d^	0.34 ± 0.02 ^b^	0.01 ± 0.00 ^d^
	Day 6	3.49 ± 0.00 ^f^	6.40 ± 0.14 ^ij^	38.71 ± 1.09 ^h^	14.13 ± 0.25 ^hi^	241.83 ± 4.01 ^f^	0.38 ± 0.02 ^bc^	0.40 ± 0.02 ^a^	0.02 ± 0.00 ^cd^
	Day 8	3.47 ± 0.00 ^f^	5.10 ± 0.00 ^l^	12.90 ± 0.56 ^j^	3.75 ± 0.11 ^j^	85.26 ± 2.33 ^h^	0.36 ± 0.02 ^c^	0.33 ± 0.01 ^b^	0.03 ± 0.01 ^bc^
	Day 10	3.42 ± 0.00 ^fg^	5.00 ± 0.00 ^l^	6.25 ± 0.69 ^k^	ND	21.75 ± 1.06 ^l^	0.32 ± 0.01 ^d^	0.27 ± 0.00 ^cd^	0.03 ± 0.01 ^bc^
	Day 12	3.35 ± 0.00 ^gh^	5.95 ± 0.07 ^jk^	ND	ND	6.64 ± 0.13 ^m^	0.37 ± 0.01 ^c^	0.31 ± 0.00 ^bc^	0.04 ± 0.00 ^ab^
	Day 14	3.28 ± 0.00 ^hi^	3.80 ± 0.28 ^m^	ND	ND	ND	0.39 ± 0.01 ^bc^	0.32 ± 0.01 ^bc^	0.05 ± 0.01 ^a^
K4	Day 0	4.17 ± 0.00 ^a^	11.25 ± 0.07 ^c^	325.08 ± 4.12 ^a^	61.23 ± 1.36 ^d^	516.01 ± 4.32 ^a^	0.28 ± 0.00 ^e^	0.16 ± 0.01 ^f^	ND
	Day 2	4.15 ± 0.01 ^ab^	11.70 ± 0.00 ^ab^	244.98 ± 3.85 ^b^	43.65 ± 1.00 ^e^	367.67 ± 2.69 ^c^	0.35 ± 0.02 ^c^	0.20 ± 0.00 ^ef^	ND
	Day 4	3.77 ± 0.01 ^d^	11.00 ± 0.00 ^c^	233.70 ± 3.03 ^b^	38.42 ± 0.19 ^f^	310.11 ± 3.39 ^d^	0.33 ± 0.01 ^d^	0.22 ± 0.01 ^e^	ND
	Day 6	3.60 ± 0.00 ^e^	9.65 ± 0.07 ^f^	192.34 ± 1.36 ^c^	36.75 ± 0.26 ^f^	95.79 ± 1.58 ^h^	0.35 ± 0.01 ^c^	0.26 ± 0.02 ^de^	ND
	Day 8	3.49 ± 0.00 ^f^	6.65 ± 0.07 ^i^	93.25 ± 1.55 ^d^	11.40 ± 0.11 ^i^	44.41 ± 1.34 ^k^	0.33 ± 0.01 ^d^	0.32 ± 0.01 ^bc^	ND
	Day 10	3.42 ± 0.01 ^fg^	6.00 ± 0.00 ^j^	38.70 ± 1.22 ^h^	5.28 ± 0.13 ^j^	ND	0.35 ± 0.01 ^c^	0.33 ± 0.00 ^b^	0.01 ± 0.00 ^d^
	Day 12	3.34 ± 0.00 ^h^	6.20 ± 0.00 ^j^	20.97 ± 0.89 ^i^	ND	ND	0.41 ± 0.02 ^b^	0.34 ± 0.02 ^b^	0.03 ± 0.00 ^bc^
	Day 14	3.27 ± 0.00 ^hi^	7.80 ± 0.12 ^h^	9.72 ± 0.46 ^k^	ND	ND	0.42 ± 0.01 ^b^	0.31 ± 0.01 ^bc^	0.04 ± 0.01 ^ab^

Means of three independent analyses ± standard deviation; values in the same columns followed by different letters (a–m) are significantly different at *p* < 0.05 according to Duncan’s test. Abbreviations: K1 (control): 100% tea; K2: 75% tea + 25% pear juice; K3: 50% tea + 50% pear juice; and K4: 25% tea + 75% pear juice.

**Table 2:** Changes in phenolic subclasses (mg/100 mL) in the tested kombucha beverages during fermentation.

		Phenolic Acids and Derivatives	Purine Alkaloids	Flavan-3-ols and Proanthocyanidins	Flavonols	Theaflavins	Flavones	Total
K1	Day 0	295.48 ± 3.13 ^a^	48.43 ± 2.58 ^d^	3166.60 ± 20.12 ^c^	394.49 ± 5.65 ^a^	66.53 ± 2.23 ^a^	ND	3971.53 ± 10.15 ^c^
	Day 2	195.68 ± 2.99 ^d^	37.34 ± 2.45 ^f^	1980.06 ± 19.68 ^f^	270.91 ± 8.12 ^b^	64.92 ± 2.09 ^b^	ND	2548.91 ± 15.28 ^g^
	Day 4	245.24 ± 3.13 ^c^	43.16 ± 2.31 ^e^	2808.75 ± 22.69 ^e^	251.77 ± 4.71 ^c^	62.81 ± 1.66 ^c^	ND	3411.73 ± 11.36 ^f^
	Day 6	243.98 ± 2.59 ^c^	46.20 ± 2.24 ^de^	2848.71 ± 20.56 ^e^	244.86 ± 6.01 ^c^	60.93 ± 2.16 ^d^	ND	3444.68 ± 12.22 ^f^
	Day 8	249.60 ± 4.25 ^c^	50.75 ± 3.49 ^cd^	2982.00 ± 15.15 ^d^	234.49 ± 3.55 ^d^	60.69 ± 2.36 ^d^	ND	3577.53 ± 15.87 ^e^
	Day 10	248.36 ± 2.09 ^c^	53.10 ± 3.00 ^c^	3181.78 ± 23.16 ^c^	231.54 ± 5.12 ^d^	58.19 ± 3.41 ^e^	ND	3772.97 ± 9.58 ^d^
	Day 12	248.98 ± 3.22 ^c^	60.56 ± 3.15 ^b^	3469.70 ± 24.52 ^b^	228.91 ± 4.87 ^e^	57.48 ± 4.18 ^f^	ND	4065.63 ± 16.84 ^b^
	Day 14	261.12 ± 3.03 ^b^	78.16 ± 2.02 ^a^	3866.89 ± 14.44 ^a^	245.38 ± 3.84 ^c^	55.89 ± 2.33 ^g^	ND	4507.44 ± 13.03 ^a^
K2	Day 0	1235.21 ± 20.48 ^b^	42.58 ± 1.52 ^c^	2294.39 ± 11.34 ^b^	751.33 ± 3.12 ^a^	50.54 ± 2.51 ^a^	40.34 ± 1.03 ^a^	4414.39 ± 20.25 ^b^
	Day 2	885.07 ± 18.48 ^f^	31.70 ± 1.21 ^e^	1593.73 ± 13.39 ^h^	683.65 ± 2.58 ^b^	46.58 ± 3.12 ^bc^	38.89 ± 1.09 ^b^	3279.62 ± 14.85 ^h^
	Day 4	975.00 ± 19.56 ^e^	36.59 ± 1.13 ^d^	1743.58 ± 16.16 ^g^	687.23 ± 4.01 ^b^	50.33 ± 1.41 ^a^	38.9 ± 0.98 ^b^	3531.63 ± 19.20 ^g^
	Day 6	981.65 ± 22.12 ^e^	39.21 ± 1.01 ^cd^	1917.05 ± 14.73 ^f^	679.25 ± 3.22 ^bc^	49.99 ± 1.62 ^a^	38.83 ± 1.05 ^b^	3705.98 ± 11.88 ^f^
	Day 8	1004.86 ± 12.76 ^d^	42.51 ± 1.24 ^c^	2063.68 ± 11.90 ^e^	658.12 ± 2.16 ^c^	47.77 ± 1.23 ^b^	38.67 ± 0.87 ^bc^	3855.61 ± 10.39 ^e^
	Day 10	1049.78 ± 20.16 ^c^	44.45 ± 0.99 ^c^	2171.02 ± 15.30 ^d^	624.63 ± 3.52 ^d^	45.66 ± 1.33 ^c^	38.45 ± 0.88 ^c^	3973.99 ± 21.09 ^d^
	Day 12	1052.88 ± 21.12 ^c^	51.05 ± 1.31 ^b^	2232.24 ± 18.27 ^c^	609.21 ± 2.84 ^e^	43.58 ± 0.89 ^cd^	38.40 ± 1.01 ^c^	4027.36 ± 17.13 ^c^
	Day 14	1376.36 ± 13.28 ^a^	65.84 ± 1.44 ^a^	2617.44 ± 16.22 ^a^	598.27 ± 3.09 ^e^	42.54 ± 1.26 ^d^	38.23 ± 0.99 ^c^	4738.68 ± 15.47 ^a^
K3	Day 0	1333.47 ± 6.45 ^b^	36.95 ± 0.24 ^b^	2491.64 ± 17.79 ^b^	907.10 ± 3.78 ^a^	37.43 ± 1.03 ^a^	52.35 ± 1.25 ^a^	4858.94 ± 14.93 ^b^
	Day 2	1197.35 ± 9.54 ^e^	30.28 ± 0.65 ^f^	2244.06 ± 17.91 ^f^	869.11 ± 2.24 ^b^	32.26 ± 0.89 ^c^	50.89 ± 2.16 ^b^	4423.95 ± 21.69 ^g^
	Day 4	1255.09 ± 0.84 ^d^	31.98 ± 0.32 ^e^	2283.54 ± 16.89 ^e^	865.04 ± 5.06 ^b^	33.25 ± 0.68 ^b^	50.84 ± 1.09 ^b^	4519.74 ± 2.526 ^f^
	Day 6	1265.14 ± 6.66 ^cd^	32.63 ± 0.28 ^de^	2365.86 ± 18.47 ^d^	852.69 ± 4.25 ^bc^	32.96 ± 1.02 ^bc^	50.67 ± 2.05 ^b^	4599.95 ± 17.75 ^e^
	Day 8	1289.96 ± 4.35 ^c^	33.82 ± 0.09 ^d^	2410.88 ± 21.049 ^c^	823.98 ± 2.64 ^c^	32.31 ± 0.02 ^c^	50.64 ± 1.33 ^b^	4641.59 ± 18.35 ^d^
	Day 10	1308.32 ± 4.74 ^bc^	34.35 ± 0.14 ^cd^	2430.36 ± 17.81 ^c^	797.58 ± 5.49 ^d^	31.60 ± 1.03 ^de^	50.50 ± 2.01 ^b^	4652.71 ± 26.79 ^cd^
	Day 12	1308.33 ± 8.97 ^bc^	34.93 ± 0.25 ^c^	2495.38 ± 18.07 ^b^	770.45 ± 5.87 ^de^	31.04 ± 0.54 ^e^	50.44 ± 2.12 ^b^	4690.57 ± 36.61 ^c^
	Day 14	1537.51 ± 4.89 ^a^	41.73 ± 0.33 ^a^	2650.44 ± 20.52 ^a^	758.85 ± 4.64 ^e^	30.24 ± 0.33 ^f^	50.40 ± 1.27 ^b^	5069.17 ± 18.02 ^a^
K4	Day 0	346.61 ± 5.34 ^a^	8.55 ± 0.51 ^c^	4368.29 ± 35.62 ^c^	1200.12 ± 22.69 ^a^	6.52 ± 0.13 ^a^	25.42 ± 0.31 ^a^	5955.51 ± 35.16 ^a^
	Day 2	317.80 ± 4.23 ^c^	8.36 ± 0.23 ^d^	4274.39 ± 25.18 ^e^	1155.72 ± 38.12 ^b^	6.17 ± 0.21 ^b^	24.87 ± 0.25 ^b^	5787.31 ± 44.85 ^e^
	Day 4	323.50 ± 6.23 ^b^	8.45 ± 0.09 ^d^	4322.92 ± 30.30 ^d^	1150.69 ± 20.25 ^b^	6.05 ± 0.18 ^b^	25.25 ± 0.16 ^a^	5836.86 ± 34.16 ^d^
	Day 6	321.91 ± 3.14 ^b^	7.38 ± 0.21 ^e^	4348.13 ± 35.48 ^cd^	1137.88 ± 18.57 ^c^	5.91 ± 0.09 ^c^	24.55 ± 0.06 ^bc^	5845.76 ± 40.55 ^cd^
	Day 8	320.84 ± 5.33 ^b^	8.49 ± 0.31 ^cd^	4377.18 ± 41.06 ^c^	1125.00 ± 36.24 ^d^	5.66 ± 0.15 ^d^	24.35 ± 0.11 ^c^	5861.52 ± 49.52 ^c^
	Day 10	316.96 ± 6.18 ^c^	8.55 ± 0.15 ^c^	4394.33 ± 15.41 ^b^	1119.46 ± 20.59 ^d^	4.83 ± 0.11 ^e^	24.08 ± 0.08 ^cd^	5868.21 ± 25.69 ^c^
	Day 12	312.24 ± 3.14 ^cd^	8.86 ± 0.14 ^b^	4396.60 ± 12.12 ^b^	1088.52 ± 15.99 ^e^	4.61 ± 0.00 ^f^	23.99 ± 0.13 ^d^	5834.82 ± 30.12 ^d^
	Day 14	310.60 ± 5.30 ^d^	9.81 ± 0.20 ^a^	4494.14 ± 16.22 ^a^	1071.57 ± 31.54 ^e^	4.55 ± 0.01 ^g^	23.90 ± 0.05 ^d^	5914.57 ± 41.57 ^b^

Means of three independent analyses ± standard deviation; values in the same columns followed by different letters (a–h) are significantly different at *p* < 0.05 according to Duncan’s test. Abbreviations: K1 (control): 100% tea; K2: 75% tea + 25% pear juice; K3: 50% tea + 50% pear juice; and K4: 25% tea + 75% pear juice.

**Table 3:** ABTS, FRAP, and DPPH antioxidant capacities [µMol Trolox/100 mL] and enzyme inhibitory potential [% of inhibition] in the tested kombucha beverages during fermentation.

	Days	ABTS	FRAP	DPPH	α-Amylase	α-Glucosidase	AChE	BuChE	COX 1	COX 2
	[µmol Trolox/100 mL]				[% of Inhibition (Concentration 1 mg/mL)]					
K1	Day 0	1581.46 ± 1.69 ^r^	786.49 ± 2.46 ^k^	910.28 ± 3.51 ^ij^	0 ± 0.00 ^d^	0 ± 0.00 ^d^	6.97 ± 0.11 ^d^	3.11 ± 0.13 ^f^	68.63 ± 4.21 ^d^	47.62 ± 2.22 ^e^
	Day 2	1015.15 ± 5.68 ^t^	368.94 ± 3.89 ^o^	490.56 ± 4.37 ^kl^						
	Day 4	2097.55 ± 4.43 ^m^	485.61 ± 5.11 ^n^	480.99 ± 5.42 ^l^						
	Day 6	1481.32 ± 3.98 ^s^	583.28 ± 6.84 ^m^	700.39 ± 2.01 ^k^						
	Day 8	1531.10 ± 6.15 ^r^	519.26 ± 4.04 ^mn^	730.11 ± 3.18 ^k^						
	Day 10	1847.00 ± 5.98 ^o^	593.65 ± 1.22 ^m^	810.37 ± 4.36 ^j^						
	Day 12	1941.91 ± 2.77 ^n^	630.44 ± 5.19 ^l^	810.81 ± 2.94 ^j^						
	Day 14	2121.28 ± 3.67 ^l^	639.29 ± 6.09 ^l^	860.92 ± 4.30 ^j^	22.15 ± 1.79 ^b^	25.01 ± 1.03 ^b^	8.08 ± 0.66 ^c^	44.85 ± 0.25 ^c^	80.39 ± 0.70 ^bc^	83.70 ± 0.63 ^b^
K2	Day 0	2469.31 ± 2.31 ^i^	872.19 ± 2.99 ^j^	1000.39 ± 5.84 ^h^	1.56 ± 0.06 ^d^	1.68 ± 0.05 ^d^	8.34 ± 0.08 ^c^	7.54 ± 0.12 ^e^	76.47 ± 0.08 ^c^	58.73 ± 0.14 ^d^
	Day 2	1004.43 ± 4.35 ^t^	648.84 ± 4.35 ^l^	1170.91 ± 4.43 ^g^						
	Day 4	1468.61 ± 5.88 ^s^	772.66 ± 7.01 ^k^	950.15 ± 7.00 ^i^						
	Day 6	1620.27 ± 6.03 ^q^	849.69 ± 6.53 ^j^	1160.23 ± 6.41 ^g^						
	Day 8	1622.29 ± 4.89 ^q^	1067.21 ± 2.97 ^h^	1240.55 ± 5.22 ^f^						
	Day 10	2027.57 ± 8.55 ^m^	806.40 ± 3.05 ^jk^	1120.76 ± 8.43 ^gh^						
	Day 12	1913.19 ± 2.31 ^n^	899.25 ± 8.09 ^j^	1190.81 ± 5.07 ^g^						
	Day 14	2594.72 ± 4.22 ^h^	999.27 ± 6.37 ^h^	1250.15 ± 4.71 ^f^	25.67 ± 1.22 ^b^	29.22 ± 0.98 ^b^	8.24 ± 0.55 ^c^	44.14 ± 2.21 ^c^	88.24 ± 5.05 ^ab^	93.40 ± 3.12 ^a^
K3	Day 0	3002.39 ± 2.63 ^e^	1154.46 ± 4.85 ^g^	1730.59 ± 4.18 ^b^	5.15 ± 0.10 ^c^	6.99 ± 0.09 ^c^	8.89 ± 0.12 ^c^	7.90 ± 0.23 ^e^	84.31 ± 5.21 ^b^	74.60 ± 4.33 ^c^
	Day 2	1477.81 ± 4.57 ^s^	719.29 ± 6.13 ^k^	900.91 ± 9.66 ^ij^						
	Day 4	1944.90 ± 2.99 ^n^	867.21 ± 8.22 ^j^	1060.00 ± 10.01 ^h^						
	Day 6	1755.00 ± 8.22 ^p^	941.66 ± 3.33 ^i^	1190.36 ± 9.34 ^g^						
	Day 8	1825.28 ± 5.21 ^o^	1002.40 ± 11.58 ^h^	1280.19 ± 8.58 ^f^						
	Day 10	2300.20 ± 6.29 ^j^	1156.00 ± 9.43 ^g^	1390.62 ± 15.58 ^e^						
	Day 12	2922.43 ± 8.93 ^f^	1415.25 ± 11.29 ^f^	1690.45 ± 10.87 ^c^						
	Day 14	3518.47 ± 10.15 ^c^	1612.92 ± 5.22 ^d^	1750.43 ± 12.79 ^b^	30.41 ± 1.03 ^ab^	30.99 ± 1.22 ^b^	11.32 ± 0.69 ^ab^	59.92 ± 1.09 ^b^	90.20 ± 2.31 ^a^	78.65 ±1.56 ^c^
K4	Day 0	3279.93 ± 9.64 ^d^	1538.41 ± 13.31 ^e^	1590.70 ± 11.98 ^d^	7.28 ± 0.12 ^c^	9.99 ± 0.54 ^c^	10.15 ± 0.31 ^b^	17.32 ± 0.17 ^d^	90.20 ± 1.32 ^a^	87.30 ± 1.52 ^b^
	Day 2	1721.00 ± 12.36 ^p^	690.40 ± 10.01 ^kl^	830.48 ± 13.31 ^j^						
	Day 4	3086.12 ± 8.15 ^e^	1061.60 ± 15.51 ^h^	1240.40 ± 16.51 ^f^						
	Day 6	2283.32 ± 14.53 ^k^	1763.24 ± 10.28 ^c^	1260.54 ± 19.19 ^f^						
	Day 8	2531.47 ± 8.86 ^h^	2301.66 ± 14.64 ^b^	1690.50 ± 16.29 ^c^						
	Day 10	2727.00 ± 13.42 ^g^	2349.6 ± 15.56 ^b^	1700.81 ± 9.99 ^bc^						
	Day 12	3651.9 ± 10.99 ^b^	2659.2 ± 10.46 ^a^	1960.33 ± 15.44 ^a^						
	Day 14	3855.47 ± 15.63 ^a^	2669.62 ± 11.89 ^a^	1970.92 ± 9.86 ^a^	35.89 ± 2.15 ^a^	40.58 ± 1.43 ^a^	13.12 ± 0.16 ^a^	73.39 ± 0.54 ^a^	94.12 ± 1.62 ^a^	95.34 ± 1.36 ^a^

Means of three independent analyses ± standard deviation; values in the same columns followed by different letters (a–t) are significantly different at *p* < 0.05 according to Duncan’s test. Abbreviations: K1 (control): 100% tea; K2: 75% tea + 25% pear juice; K3: 50% tea + 50% pear juice; and K4: 25% tea + 75% pear juice.

## Data Availability

Data will be made available upon request.

## Supplementary Materials

The following supporting information can be downloaded at: https://www.mdpi.com/article/10.3390/molecules31020371/s1, Table S1: Retention times and characteristic ions of phenolic compounds in tested kombucha beverages; Table S2: The content of phenolic compounds (mg/100 mL) in kombucha 1 (K1) during fermentation; Table S3: The content of phenolic compounds (mg/100 mL) in kombucha 2 (K2) during fermentation; Table S4: The content of phenolic compounds (mg/100 mL) in kombucha 3 (K3) during fermentation; Table S5: The content of phenolic compounds (mg/100 mL) in kombucha 4 (K4) during fermentation.
