# Supplementary material for: Dose-Dependent Effects of Pear (Pyrus communis L.) Juice on Kombucha Polyphenols, Antioxidant Capacity, and Enzyme Inhibition
Source: Molecules. 2026 Jan 20;31(2):371. doi: 10.3390/molecules31020371 (PMC12844145; doi:10.3390/molecules31020371)
Supplement: Supplementary file 1 [file molecules-31-00371-s001.zip › molecules-4075711-supplementary.pdf]

Supplementary Table S1. Retention times and characteristic ions of phenolic compounds in tested kombucha beverages.

Rt (min)	[M–H]⁻ (m/z)[1]	MS/MS Fragments (m/z)[1]	Tentative Identification	K1	K2	K3	K4
0.73	341.0559	191.9807	Caffeoylhexoside		√	√	√
0.93	331.0069	169.0545	Galloylhexose isomer	√	√	√	√
1.27	173.0584	155.9472	Theanine	√	√	√	√
1.40	341.0559	191.0312	Caffeoylhexoside				√
1.50	343.9927	191.0108	Theogallin	√	√	√	√
1.63	331.0143	168.9768	Galloylhexose isomer	√	√	√	√
1.83	687.0665	343.0159/191.0167	Theogallin dimer	√	√	√	√
2.03	341.0465	173.0659	Coffeoylhexose		√	√	√
2.19	341.0219	191.0149	Caffeoylhexoside	√	√	√	√
2.33	343.0116	169.0762	3-galloylquinic acid	√	√	√	√
2.61	179.0066	136.0561	Theobromine	√	√	√	√
2.65	305.0150	291.0288/173.9810	(+)-Gallocatechin	√	√	√	
2.98	353.0136	191.0553/179.2167	*cis*-3-Caffeoylquinic acid		√	√	√
3.09	609.1352	305.0691/197.1232	Gallocatechin dimer unknown isomer	√	√	√	
3.22	353.0315	191.0203/178.9963	5-Caffeoylquinic acid	√	√	√	√
3.35	365.0702	229.0458	Caffeoyl-N-tryptophan				√
3.42	609.0455	305.0691/197.1232	Gallocatechin dimer unknown isomer	√	√	√	
3.76	611.0702	305.0167	(+)-Gallocatechin dimer	√	√	√	√
3.85	305.0146	219.8142/191.01258/173.0330	(-)-Epigallocatechin	√	√	√	√
3.93	337.0395	163.0036	*cis*-4-*p*-Coumaroylquinic acid	√	√	√	√
4.12	331.0276	168.9721	Galloylhexose isomer	√	√	√	
4.13	353.0320	191.0145	Caffeoylquinic acid				√
4.20	289.0235	245.0244/124.9910	(+)-Catechin	√	√	√	√
4.26	579.0485	289.0157	B-type procyanidin dimer		√	√	√
4.31	511.0567	365.1265/265.433	Caffeoyl-N-tryptophanrhamnoside	√	√	√	√
4.41	633.0020	300.9991/275.0238	(-)-Methylepigallocatechin gallate	√	√	√	√
4.58	577.0702	289.0255	B-type procyanidin dimer				√
4.67	577.0524	289.0271	B-type procyanidin dimer		√	√	√
4.77	577.0633	289.0255	B-type procyanidin dimer				√
4.80	913.0565	591.0791/168.9979	Theasinensins	√			
4.87	865.1355	577.1325/289.1277	Procyanidin trimer isomer	√	√	√	√
5.06	337.0400	191.0154	5-*p*-Coumaroylquinic acid	√	√	√	√
5.10	289.0230	245.0160	(-)-Epicatechin	√	√	√	√
5.20	915.0837	457.0162/168.9780	(-)-Epigallocatechin gallate	√	√	√	√

5.32	337.0393	173.0079	3-*p*-Coumaroylquinic acid		√	√	√
5.47	479.0205	315.9629	Mirycetin glucoside	√	√	√	√
5.54	169.0764	124.9914	Gallic acid	√	√	√	√
5.61	457.0184	305.0655/168.9764	Gallocatechin gallate isomer	√	√	√	√
5.75	635.0045	483.0208/313.0050	Trigalloylhexose	√	√	√	√
5.83	563.0670	443.0061/315.1022	Theaflavin isomer	√	√	√	√
5.86	563.0727	443.0061/315.1023	Theaflavin isomer	√	√	√	√
5.95	479.0211	315.9221	Mirycetin galactoside	√	√	√	√
6.10	771.1214	301.0245	Quercetin-3-*O*-glucosylrutinoside	√	√	√	√
6.24	593.0818	285.0063	Kaempferol 3-*O*-*p*-coumaroylhexoside isomer	√	√	√	√
6.31	771.1218	301.0245	Quercetin-3-*O*-glucosylrutinoside	√	√	√	√
6.41	755.1249	300.9985	Quercetin 3-*O*-dirhamnosylhexoside	√	√	√	√
6.57	577.0834	289.0685	B-type procyanidin dimer		√	√	√
6.59	609.0764	301.0231	Quercetin hexoside-deoxyhexoside	√	√	√	√
6.70	463.0264	301.0402	Quercetin 3-*O*-glucoside	√	√	√	√
6.79	441.0225	289.0226	(+)-Epicatechin gallate	√	√	√	√
6.83	463.0260	301.0264	Quercetin 3-*O*-galactoside	√	√	√	√
6.96	755.1271	285.0410	Kaempferol 3-*O*-*p*-glucosylrutinoside isomer	√	√	√	√
7.21	739.1308	593.1550/285.9912	Kaempferol 3-*O*-2'',6''-di-*p*-coumaroylglucoside	√	√	√	√
7.31	593.0286	549.2916/357.1320/285.0762	Kaempferol 3-*O*-rutinoside isomer	√	√	√	√
7.47	623.0826	315.0442	Isorhamnetin 3-*O*-rutinoside				√
7.57	447.0329	284.9989	Kaempferol 3-*O*-glucoside	√	√	√	√
7.79	447.0446	285.8467	Kaempferol 3-*O*-galactoside	√	√	√	√
8.20	789.0946	305.0127	Gallocatechin unknown derivative	√	√	√	
8.82	627.0617	315.9769	Isorhamnetin derivtive				√
9.16	1049.1871	300.9903	Quercetin 3-*O*-acylglycoside isomer	√	√	√	√
9.31	1033.1851	284.9967	Kaempferol 3-*O*-*p*-coumaroylrhamnosyldihexoside isomer	√	√	√	
9.39	887.1379	301.0877	Quercetin 3-*O*-*p*-coumaroyldirhamnosylhexoside isomer	√	√	√	√
9.53	563.0520	269.0039	Apigenin *C*-hexoside-*C*-pentoside		√	√	√
9.59	1033.1943	284.9887	Kaempferol 3-*O*-p-coumaroylrhamnosyldihexoside isomer	√	√	√	√
9.92	1033.2047	284.9918	Kaempferol 3-*O*-p-coumaroylrhamnosyldihexoside isomer		√	√	
9.99	519.1157	315.2156	Isorhamnetin-acylated-hexoside		√	√	√
10.24	519.0578	315.0456	Isorhamnetin-acylated-hexoside		√	√	√

[1] Experimental data. [2] Identified using corresponding authentic standards

Supplementary Table S2. The content of phenolic compounds (mg/100 mL) in kombucha 1 (K1) during fermentation

Compound		Day 0	Day 2	Day 4	Day 6	Day 8	Day 10	Day 12	Day 14
**Phenolic acids and derivatives**									
Quinic acid		1.15 ±0.11^{a}	0.88 ±0.11^{c}	1.03 ±0.08^{ab}	1.00 ±0.02^{ab}	0.98 ±0.01^{b}	0.98 ±0.01^{b}	0.76 ±0.01^{d}	0.57 ±0.01^{e}
Galloylhexose isomer		0.19 ±0.01^{a}	0.09 ±0^{b}	0.02 ±0^{d}	0.00 ±0.00^{d}	0.00 ±0.00^{d}	0.00 ±0.00^{d}	0.00 ±0.00^{d}	0.00 ±0.00^{d}
Theogallin		13.08 ±0.51^{d}	11.36 ±0.31^{e}	15.89 ±0.33^{a}	15.52 ±0.25^{ab}	15.21 ±0.13^{bc}	15.07 ±0.3^{c}	14.99 ±0.18^{c}	14.99 ±0.24^{c}
Galloylhexose isomer		41.13 ±1^{a}	28.49 ±0.42^{c}	37.63 ±0.29^{b}	28.18 ±0.22^{c}	20.43 ±0.14^{d}	12.15 ±0.09^{e}	5.10 ±0.01^{f}	0.11 ±0.00^{g}
Theogallin dimer		114.48 ±2.5^{a}	83.96 ±1.21^{d}	98.67 ±1.66^{c}	100.25 ±1.58^{bc}	105.05 ±1.79^{b}	105.49 ±1.68^{b}	106.56 ±2.01^{b}	116.37 ±1.99^{a}
Caffeoylhexoside		3.09 ±0.05^{c}	2.34 ±0.01^{d}	3.05 ±0.16^{c}	3.09 ±0.09^{c}	3.11 ±0.11^{c}	3.23 ±0.07^{b}	3.30 ±0.09^{ab}	3.39 ±1.00^{a}
3-galloylquinic acid		1.82 ±0.02^{a}	0.86 ±0.01^{f}	1.60 ±0.09^{b}	1.62 ±0.05^{b}	1.48 ±0.09^{c}	1.27 ±0.1^{d}	1.10 ±0.05^{e}	1.02 ±0.02^{e}
5-Caffeoylquinic acid		10.51 ±0.36^{a}	6.73 ±0.13^{f}	7.73 ±0.54^{de}	7.06 ±0.44^{e}	6.86 ±0.52^{f}	7.34 ±0.29^{e}	8.00 ±0.31^{c}	9.18 ±0.19^{b}
cis-4-p-Coumaroylquinic acid		34.78 ±0.58^{bc}	22.01 ±0.25^{d}	24.88 ±0.66^{d}	25.15 ±0.19^{d}	30.34 ±0.58^{c}	35.17 ±0.56^{bc}	39.45 ±0.48^{b}	44.43 ±0.97^{a}
Galloylhexose isomer		5.96 ±0.09^{a}	3.84 ±0.12^{c}	5.16 ±0.18^{b}	4.89 ±0.21^{b}	4.13 ±0.11^{c}	3.39 ±0.00^{d}	3.00 ±0.22^{d}	2.67 ±0.09^{e}
Caffeoyl-N-tryptophanrhamnoside		3.20 ±0.03^{h}	3.47 ±0.06^{g}	6.35 ±0.19^{c}	7.12 ±0.09^{a}	6.72 ±0.11^{b}	5.90 ±0.05^{d}	5.15 ±0.08^{e}	4.77 ±0.01^{f}
5-p-Coumaroylquinic acid		13.11 ±0.09^{d}	10.84 ±0.11^{e}	12.91 ±0.87^{d}	13.24 ±0.15^{cd}	13.67 ±0.32^{c}	14.04 ±0.58^{b}	14.48 ±0.01^{ab}	14.74 ±0.11^{a}
Gallic acid		33.02 ±0.12^{c}	11.08 ±0.41^{e}	23.23 ±1.06^{d}	30.30 ±0.89^{c}	35.61 ±0.28^{c}	39.19 ±1.15^{b}	42.30 ±0.99^{ab}	44.98 ±1.02^{a}
Trigalloylhexose		19.96 ±0.52^{a}	9.73 ±0.28^{b}	7.09 ±0.51^{c}	6.56 ±0.48^{d}	6.01 ±0.62^{e}	5.14 ±0.33^{f}	4.79 ±0.28^{g}	3.90 ±0.27^{h}
	SUM	295.48 ±10.37^{a}	195.68 ±5.94^{d}	245.24 ±11.47^{c}	243.98 ±8.07^{c}	249.60 ±8.33^{c}	248.36 ±9.02^{c}	248.98 ±8.18^{c}	261.12 ±10.25^{b}
**Flavan-3-ols and Proanthocyanidins**									
(+)-Gallocatechin		16.19 ±1.23^{b}	9.59 ±1.11^{d}	13.70 ±0.58^{c}	14.00 ±1.36^{c}	15.87±1.48^{a}	17.17 ±1.2^{b}	19.23 ±1.09^{a}	22.61 ±1.41^{a}
Gallocatechin dimer unknown isomer		1.72 ±0.52^{a}	0.97 ±0.23^{d}	1.39 ±0.44^{c}	1.39 ±0.11^{c}	1.42 ±0.09^{c}	1.41 ±0.23^{c}	1.45 ±0.13^{bc}	1.49 ±0.25^{b}
Gallocatechin dimer unknown isomer		2.26 ±0.26^{a}	1.31 ±0.22^{d}	1.11 ±0.13f^{g}	1.08 ±0.24^{g}	1.15 ±0.12^{f}	1.20 ±0.1^{e}	1.39 ±0.09^{c}	1.73 ±0.15^{b}
(+)-Gallocatechin dimer		63.37 ±2.52^{b}	41.15 ±2.32^{e}	53.12 ±4.15^{cd}	52.14 ±1.99^{d}	55.00 ±2.36^{c}	59.37 ±3.69^{bc}	63.89 ±4.15^{b}	73.11 ±5.33^{a}
(-)-Epigallocatechin		5.90 ±0.66^{a}	3.34 ±0.25^{f}	5.23 ±0.45^{c}	5.34 ±0.39^{bc}	5.46 ±0.55^{b}	5.23 ±0.09^{c}	4.89 ±0.17^{d}	4.32 ±0.52^{e}
(+)-Catechin		1695.32 ±12.59^{c}	1017.34 ±15.15^{f}	1530.47 ±20.22^{e}	1522.76 ±17.65^{e}	1578.34 ±10.29^{d}	1699.91 ±20.00^{c}	1873.12 ±18.56^{b}	2087.56 ±11.58^{a}
(-)-Methylepigallocatechin gallate		45.98 ±2.15^{a}	32.16 ±3.23^{b}	45.96 ±3.22^{a}	46.16 ±4.18^{a}	45.81 ±2.19^{a}	44.87 ±2.58^{a}	44.03 ±3.65^{a}	43.84 ±4.09^{a}
Theasinensins		15.48 ±0.59^{b}	6.45 ±0.66^{f}	11.94 ±0.35^{e}	12.36 ±0.81^{d}	12.98 ±0.21^{d}	14.01 ±0.19^{c}	15.62 ±0.3^{b}	17.12 ±0.25^{a}
Procyanidin trimer isomer		31.53 ±2.56^{a}	10.50 ±0.77^{b}	7.87 ±0.63^{c}	7.41 ±0.27^{cd}	6.99 ±0.12^{d}	6.53 ±0.36^{d}	5.78 ±0.21^{e}	5.69 ±0.19^{e}
(-)-Epicatechin		171.47 ±2.52^{a}	125.09 ±3.12^{e}	133.09 ±1.66^{de}	137.78 ±2.85^{d}	142.52 ±3.45^{cd}	149.80 ±2.69^{c}	153.72 ±3.88^{bc}	157.88 ±4.40^{b}
(-)-Epigallocatechin gallate		879.53 ±5.65^{c}	525.88 ±9.18^{g}	697.60 ±6.55^{f}	734.81 ±4.14^{e}	799.35 ±8.55^{d}	850.12 ±5.45^{c}	946.72 ±9.33^{b}	1099.40 ±6.28^{a}
Gallocatechin gallate isomer		4.69 ±0.23^{a}	2.94 ±0.11^{d}	3.12 ±0.09^{c}	3.25 ±0.12^{c}	3.33 ±0.15^{bc}	3.48 ±0.08^{b}	4.52 ±0.20^{a}	3.64 ±0.16^{b}
(+)-Epicatechin gallate		233.16 ±2.12^{c}	203.34 ±2.86^{d}	304.15 ±4.51^{b}	310.23 ±2.66^{b}	311.13 ±3.29^{b}	322.56 ±4.14^{a}	325.00 ±2.08^{a}	333.56 ±3.99^{a}
Gallocatechin unknown derivative		0.00 ±0.00^{e}	0.00 ±0.00^{e}	0.00 ±0.00^{e}	0.00 ±0.00^{e}	2.65 ±0.05^{d}	6.12 ±0.09^{c}	10.34 ±0.12^{b}	14.94 ±0.10^{a}
	SUM	3166.60 ±58.20^{c}	1980.06 ±67.91^{f}	2808.75 ±74.44^{e}	2848.71 ±63.69^{e}	2982.00 ±56.98^{d}	3181.78 ±70.82^{c}	3469.70 ±76.14^{b}	3866.89 ±67.03^{a}
**Flavonols**									
Mirycetin glucoside		3.09 ±0.23^{a}	3.06 ±0.16^{ab}	3.04 ±0.23^{b}	3.04 ±0.2^{b}	3.02 ±0.21^{bc}	2.98 ±0.12^{c}	2.25 ±0.10^{d}	2.19 ±0.15^{d}

Mirycetin galactoside	16.17 ±2.01^{a}	13.45 ±1.56^{b}	7.13 ±1.23^{c}	6.34 ±1.22^{cd}	5.12 ±1.42^{de}	4.45 ±1.05^{ef}	3.39 ±1.13^{g}	3.12 ±0.89^{g}
Quercetin-3-O-glucosylrutinoside	46.49 ±3.65^{a}	15.17 ±1.28^{e}	30.17 ±2.13^{b}	29.98 ±2.11^{bc}	27.13 ±0.85^{c}	27.01 ±2.26^{c}	25.34 ±1.28^{d}	33.96 ±2.51^{b}
Kaempferol 3-O-p-coumaroylhexoside isomer	17.35 ±1.23^{a}	10.12 ±0.69^{b}	7.98 ±1.22^{c}	7.87 ±1.030^{c}	7.65 ±1.32^{c}	7.01 ±1.12^{d}	5.99 ±1.09^{e}	4.18 ±1.44^{f}
Quercetin-3-O-glucosylrutinoside	87.62 ±3.65^{a}	77.58 ±4.15^{b}	55.42 ±2.85^{c}	50.54 ±3.12^{cd}	45.18 ±2.59^{d}	42.36 ±3.33^{d}	41.14 ±4.16^{de}	40.27 ±2.87^{e}
Quercetin 3-O-dirhamnosylhexoside	8.18 ±0.13^{a}	6.20 ±0.19^{b}	6.00 ±0.55^{b}	5.73 ±0.12^{c}	5.32 ±0.69^{d}	5.01 ±0.36^{de}	4.89 ±0.36^{e}	4.79 ±0.20^{e}
Quercetin hexoside-deoxyhexoside	87.36 ±2.15^{a}	71.73 ±1.69^{b}	66.68 ±3.01^{c}	66.15 ±2.55^{c}	65.98 ±1.68^{c}	65.45 ±2.84^{c}	65.00 ±1.56^{c}	65.01 ±1.20^{c}
Quercetin 3-O-glucoside	28.93 ±1.03^{a}	15.56 ±1.25^{b}	13.20 ±1.06^{c}	12.21 ±0.69^{d}	11.89 ±1.03^{de}	11.65 ±0.96^{e}	11.21 ±0.85^{f}	12.99 ±0.65^{c}
Quercetin 3-O-galactoside	12.42 ±1.03^{e}	11.34 ±1.05^{f}	17.95 ±0.66^{a}	16.98 ±0.98^{c}	16.56 ±1.03^{d}	16.11 ±1.00^{d}	16.56 ±0.45^{d}	17.46 ±0.39^{b}
Kaempferol 3-O-p-glucosylrutinoside isomer	26.77 ±1.25^{c}	7.83 ±0.23^{g}	16.12 ±0.56^{f}	19.82 ±0.99^{e}	21.36 ±0.54^{d}	25.51 ±0.62^{c}	30.33 ±0.23^{b}	39.77 ±0.01^{a}
Kaempferol 3-O-2",6"-di-p-coumaroylglucoside	2.70 ±0.08^{a}	1.29 ±0.10^{e}	1.53 ±0.06^{d}	1.55 ±0.03^{d}	1.60 ±0.02^{cd}	1.62 ±0.02^{c}	1.75 ±0.03^{b}	1.85 ±0.04^{b}
Kaempferol 3-O-rutinoside isomer	13.36 ±0.25^{a}	11.58 ±0.63^{b}	6.46 ±0.13^{c}	6.01 ±0.09^{d}	5.66 ±0.02^{e}	5.46 ±0.05^{e}	5.12 ±0.00^{f}	5.08 ±0.021^{g}
Kaempferol 3-O-glucoside	4.08 ±0.33^{a}	3.58 ±0.15^{b}	0.94 ±0.06^{c}	0.86 ±0.10^{cd}	0.80 ±0.00^{d}	0.72 ±0.02^{e}	0.66 ±0.01^{f}	0.60 ±0.00^{g}
Kaempferol 3-O-galactoside	6.70 ±0.32^{a}	5.43 ±0.10^{b}	4.03 ±0.01^{c}	3.95 ±0.11^{c}	3.67 ±0.19^{cd}	3.53 ±0.20^{de}	3.33 ±0.10^{e}	3.19 ±0.13^{f}
Quercetin 3-O-acylglycoside isomer	19.30 ±0.36^{a}	10.95 ±0.58^{b}	8.02 ±0.24^{c}	7.06 ±0.32^{d}	7.00 ±0.29^{d}	6.58 ±0.20^{e}	6.16 ±0.15^{f}	6.04 ±0.26^{f}
Kaempferol 3-O-p-coumaroylrhamnosyldihexoside isomer	2.10 ±0.12^{a}	0.12 ±0.01^{f}	0.79 ±0.02^{b}	0.76 ±0.01^{b}	0.72 ±0.02^{b}	0.50 ±0.01^{c}	0.34 ±0.01^{d}	0.23 ±0.02^{e}
Quercetin 3-O-p-coumaroyldirhamnosylhexoside isomer	4.72 ±0.21^{a}	2.43 ±0.13^{e}	3.81 ±0.18^{b}	3.80 ±0.24^{b}	3.77 ±0.12^{bc}	3.65 ±0.15^{c}	3.60 ±0.10^{cd}	3.54 ±0.09^{d}
Kaempferol 3-O-p-coumaroylrhamnosyldihexoside isomer	7.15 ±0.18^{a}	3.49 ±0.20^{b}	2.50 ±0.15^{c}	2.21 ±0.14^{d}	2.06 ±0.19^{de}	1.94 ±0.12^{e}	1.85 ±0.11^{e}	1.11 ±0.01^{f}
SUM	394.49 ±31.54^{a}	270.91 ±24.51^{b}	251.77 ±24.85^{c}	244.86 ±24.34^{c}	234.49 ±21.15^{d}	231.54 ±24.99^{d}	228.91 ±20.30^{e}	245.38 ±18.85^{c}
**Purine Alkaloids**								
Theanine	2.15 ±0.06^{f}	3.73 ±0.14^{e}	4.05 ±0.17^{d}	4.06 ±0.09^{d}	4.76 ±0.14^{c}	4.89 ±0.09^{b}	4.94 ±0.11^{ab}	5.15 ±0.16^{a}
Theobromine	46.28 ±2.15^{cd}	33.61 ±1.98^{g}	39.11 ±2.03^{f}	42.14 ±2.14^{e}	45.99 ±3.26^{d}	48.21 ±2.58^{c}	55.62 ±3.06^{b}	73.01 ±1.22^{a}
SUM	48.43 ±3.83^{d}	37.34 ±3.67^{f}	43.16 ±3.81^{e}	46.20 ±3.86^{de}	50.75 ±5.89^{cd}	53.10 ±4.62^{c}	60.56 ±5.49^{b}	78.16 ±2.39^{a}
**Theaflavins**								
Theaflavin isomer	49.77 ±1.36^{a}	48.79 ±1.28^{ab}	46.82 ±1.30^{bc}	45.15 ±2.03^{c}	45.09 ±2.15^{c}	42.67 ±3.15^{de}	41.98 ±4.01^{ef}	40.40 ±2.09^{f}
Theaflavin isomer	16.76 ±0.12^{a}	16.13 ±0.10^{b}	15.99 ±0.09^{bc}	15.78 ±0.11^{cd}	15.60 ±0.14^{de}	15.52 ±0.08^{e}	15.50 ±0.12^{e}	15.49 ±0.03^{e}
SUM	66.53 ±2.56^{a}	64.92 ±2.39^{b}	62.81 ±2.41^{c}	60.93 ±3.71^{d}	60.69 ±3.97^{d}	58.19 ±5.59^{e}	57.48 ±7.15^{f}	55.89 ±3.67^{g}
TOTAL	3971.53 ±106.50^{c}	2548.91 ±104.43^{g}	3411.73 ±116.98^{f}	3444.68 ±103.66^{f}	3577.53 ±96.32^{e}	3772.97 ±115.06^{d}	4065.63 ±117.26^{b}	4507.44 ±102.19^{a}

Means of three separate analyses ± standard deviation. Duncan's test reveals significant differences ($p < 0.05$) between values in the same rows with different letters (a-h).

Supplementary Table S3. The content of phenolic compounds (mg/100 mL) in kombucha 2 (K2) during fermentation

Compound	Day 0	Day 2	Day 4	Day 6	Day 8	Day 10	Day 12	Day 14
**Phenolic acids and derivatives**								
Quinic acid	1.95 ±0.12[a]	1.70 ±0.03[d]	1.80 ±0.15[c]	1.85 ±0.14[bc]	1.79 ±0.12[c]	1.75 ±0.06[cd]	1.60 ±0.08[e]	1.50 ±0.01[f]
Caffeoylhexoside	0.92 ±0.02[a]	0.50 ±0.03[bc]	0.46 ±0.02[d]	0.53 ±0.02[b]	0.48 ±0.03[cd]	0.48 ±0.01[cd]	0.53 ±0.02[b]	0.55 ±0.01[b]
Galloylhexose isomer	0.23 ±0.02[a]	0.20 ±0.01[a]	0.15 ±0.01[b]	0.09 ±0.00[c]	0.00 ±0.00[d]	0.00 ±0.00[d]	0.00 ±0.00[d]	0.00 ±0.00[d]
Theogallin	10.86 ±0.98[e]	5.15 ±0.26[g]	10.35 ±0.32[f]	11.23 ±0.84[d]	12.45 ±0.56[c]	12.80 ±0.74[b]	12.88 ±0.62[ab]	13.01 ±0.13[a]
Galloylhexose isomer	35.66 ±1.12[a]	30.32 ±0.99[b]	31.15 ±2.15[b]	27.53 ±1.69[c]	24.13 ±1.36[d]	20.20 ±1.21[e]	14.54 ±1.20[f]	10.83 ±1.03[g]
Theogallin dimer	100.45 ±3.59[ab]	84.66 ±2.58[d]	98.78 ±3.15[c]	99.56 ±4.41[bc]	99.99 ±2.84[b]	101.21 ±3.12[a]	103.12 ±4.61[a]	98.91 ±2.56[c]
Coffeoylhexose	17.23 ±1.06[b]	9.98 ±0.99[e]	18.65 ±1.03[a]	17.67 ±1.14[b]	17.01 ±1.05[bc]	16.88 ±1.02[c]	16.53 ±0.85[cd]	16.43 ±0.33[d]
Caffeoylhexoside	3.35 ±0.21[b]	2.61 ±0.22[d]	3.12 ±0.11[c]	3.15 ±0.10[c]	3.17 ±0.20[c]	2.76 ±0.19[d]	3.46 ±0.25[a]	3.51 ±0.12[a]
3-galloylquinic acid	1.63 ±0.08[a]	1.01 ±0.02[e]	1.34 ±0.03[bc]	1.39 ±0.00[b]	1.27 ±0.01[c]	1.11 ±0.02[d]	1.00 ±0.01[e]	0.87 ±0.00[f]
cis-3-Caffeoylquinic acid	15.58 ±1.36[a]	10.23 ±1.22[f]	11.23 ±1.09[e]	12.87 ±1.33[d]	13.01 ±1.08[cd]	13.46 ±1.21[c]	13.79 ±1.15[bc]	14.00 ±1.05[b]
5-Caffeoylquinic acid	10.05 ±0.36[a]	6.42 ±0.21[f]	7.33 ±0.16[c]	6.79 ±0.30[e]	6.89 ±0.25[de]	6.91 ±0.31[d]	7.12 ±0.15[cd]	7.80 ±0.10[b]
cis-4-p-Coumaroylquinic acid	39.45 ±1.06[ab]	18.70 ±0.98[d]	31.67 ±0.58[c]	31.45 ±0.29[c]	34.57 ±0.61[bc]	39.67 ±0.39[ab]	42.18 ±0.58[a]	37.77 ±0.91[b]
Galloylhexose isomer	4.97 ±0.10[a]	2.12 ±0.15[e]	4.36 ±0.26[b]	4.22 ±0.30[b]	4.01 ±0.15[c]	3.98 ±0.18[c]	3.12 ±0.10[d]	3.01 ±0.20[d]
Caffeoyl-N-tryptophanrhamnoside	2.62 ±0.16[g]	2.90 ±0.14[f]	3.19 ±0.09[ef]	3.25 ±0.15[de]	3.32 ±0.13[cd]	3.39 ±0.20[bc]	3.41 ±0.05[ab]	3.50 ±0.14[a]
5-p-Coumaroylquinic acid	46.80 ±2.51[a]	28.33 ±1.84[b]	26.68 ±2.02[bc]	26.26 ±1.65[bc]	25.94 ±1.13[cd]	25.25 ±1.47[cd]	25.01 ±1.53[cd]	24.30 ±1.09[d]
3-p-Coumaroylquinic acid	900.87 ±10.33[b]	654.79 ±15.15[f]	692.31 ±10.68[e]	700.04 ±11.62[e]	721.56 ±12.54[de]	732.86 ±14.89[d]	766.12 ±19.65[cd]	1100.86 ±18.22[a]
Gallic acid	25.56 ±1.10[c]	10.12 ±1.02[g]	15.56 ±1.32[f]	20.45 ±1.15[e]	22.98 ±1.48[d]	31.22 ±1.26[b]	33.32 ±1.08[b]	36.30 ±1.33[a]
Trigalloylhexose	17.03 ±0.52[a]	15.33 ±0.30[b]	16.87 ±0.15[a]	13.32 ±0.21[c]	12.29 ±0.15[d]	10.98 ±0.30[e]	5.15 ±0.08[f]	3.21 ±0.19[g]
SUM	1235.21 ±42.78[b]	885.07 ±45.28[f]	975.00 ±40.39[e]	981.65 ±43.89[e]	1004.86 ±41.03[d]	1049.78 ±46.04[c]	1052.88 ±55.44[c]	1376.36 ±47.49[a]
Flavan-3-ols and Proanthocyanidins								
(+)-Gallocatechin	13.99 ±2.03[d]	8.38 ±1.28[g]	11.88 ±0.98[f]	12.13 ±1.13[e]	13.72 ±1.25[d]	14.82 ±1.10[c]	16.58 ±0.99[b]	19.45 ±1.06[a]
Gallocatechin dimer unknown isomer	1.42 ±0.30[a]	0.90 ±0.06[d]	1.29 ±0.10[c]	1.29 ±0.09[c]	1.32 ±0.02[bc]	1.33 ±0.10[bc]	1.35 ±0.08[b]	1.27 ±0.10[c]
Gallocatechin dimer unknown isomer	1.90 ±0.06[a]	1.10 ±0.03[cd]	0.91 ±0.08[e]	0.86 ±0.02[e]	1.01 ±0.00[d]	1.12 ±0.06[c]	1.15 ±0.03[bc]	1.21 ±0.02[b]
(+)-Gallocatechin dimer	53.86 ±1.13[ab]	35.15 ±1.25[g]	42.18 ±1.41[f]	43.39 ±1.09[ef]	45.55 ±1.16[de]	47.82 ±1.32[cd]	50.32 ±1.02[bc]	55.30 ±1.52[a]
(-)-Epigallocatechin	5.15 ±0.16[a]	3.21 ±0.12[g]	3.89 ±0.08[f]	4.12 ±0.12[e]	4.56 ±0.04[d]	4.60 ±0.32[cd]	4.62 ±0.05[c]	4.78 ±0.06[b]
(+)-Catechin	1720.45 ±6.88[b]	1121.15 ±9.54[h]	1235.84 ±3.81[g]	1388.13 ±8.52[f]	1512.34 ±10.10[e]	1598.00 ±12.45[d]	1650.46 ±6.79[c]	2014.56 ±8.43[a]
B-type procyanidin dimer	23.45 ±1.52[a]	13.13 ±0.56[b]	10.23 ±1.06[cd]	9.88 ±1.02[de]	9.43 ±0.59[ef]	9.21 ±0.46[fg]	9.09 ±0.47[gh]	8.89 ±0.80[h]
(-)-Methylepigallocatechin gallate	40.08 ±1.03[bc]	29.92 ±0.58[e]	40.22 ±0.62[b]	39.98 ±0.28[c]	41.12 ±0.39[a]	40.56 ±0.30[b]	39.09 ±0.45[d]	39.00 ±0.84[d]
B-type procyanidin dimer	29.43 ±1.06[a]	28.99 ±1.11[ab]	28.67 ±1.06[b]	27.98 ±0.98[c]	27.15 ±0.15[cd]	26.66 ±1.25[d]	26.54 ±1.31[de]	26.13 ±0.58[e]
Procyanidin trimer isomer	15.15 ±0.29[a]	10.44 ±0.25[f]	10.87 ±0.17[ef]	10.98 ±0.32[e]	11.02 ±0.16[de]	11.56 ±0.23[d]	12.21 ±0.30[c]	13.55 ±0.08[b]
(-)-Epicatechin	11.0 ±0.55[d]	9.90 ±0.32[f]	10.90 ±0.25[e]	10.99 ±0.16[d]	11.21 ±0.20[c]	11.65 ±0.18[bc]	11.98 ±0.32[a]	12.05 ±0.40[a]
(-)-Epigallocatechin gallate	143.67 ±2.65[a]	112.54 ±2.99[f]	113.12 ±3.05[ef]	115.23 ±1.65[e]	119.34 ±3.12[de]	123.43 ±2.58[cd]	128.67 ±2.44[bv]	130.62 ±3.57[b]
Gallocatechin gallate isomer	4.00 ±0.08[a]	3.50 ±0.12[b]	3.48 ±0.13[b]	3.21 ±0.10[bc]	3.01 ±0.09[c]	2.95 ±0.12[cd]	2.90 ±0.10[d]	3.09 ±0.06[c]
B-type procyanidin dimer	30.66 ±0.28[a]	25.45 ±0.36[d]	28.67 ±0.21[b]	28.32 ±0.40[b]	28.06 ±0.36[b]	27.79 ±0.30[c]	27.51 ±0.29[c]	27.31 ±0.35[c]
(+)-Epicatechin gallate	200.18 ±1.33[e]	189.97 ±2.06[f]	201.43 ±1.54[e]	220.56 ±1.06[d]	233.71 ±1.58[c]	247.98 ±1.22[b]	247.99 ±1.61[b]	258.35 ±1.09[a]

Gallocatechin unknown derivative	0.00 ±0.00^{e}	0.00 ±0.00^{e}	0.00 ±0.00^{e}	0.00 ±0.00^{e}	1.13 ±0.01^{d}	1.54 ±0.01^{c}	1.78 ±0.02^{b}	1.88 ±0.02^{a}
SUM	2294.39 ±33.52^{b}	1593.73 ±35.73^{h}	1743.58 ±25.20^{g}	1917.05 ±29.34^{f}	2063.68 ±33.29^{e}	2171.02 ±38.11^{d}	2232.24 ±28.18^{c}	2617.44 ±32.87^{a}
**Flavonols**								
Mirycetin glucoside	1.15 ±0.06^{a}	1.05 ±0.02^{ab}	0.97 ±0.03^{bc}	0.67 ±0.03^{cd}	0.41 ±0.02^{de}	0.20 ±0.01^{e}	0.00 ±0.00^{f}	0.00 ±0.00^{f}
Mirycetin galactoside	3.74 ±0.12^{a}	1.43 ±0.09^{b}	1.06 ±0.07^{d}	1.38 ±0.03^{bc}	1.35 ±0.05^{c}	0.78 ±0.02^{ef}	0.60 ±0.03^{fg}	0.55 ±0.02^{g}
Quercetin-3-O-glucosylrutinoside	74.51 ±0.28^{a}	47.12 ±0.36^{c}	60.67 ±0.41^{b}	60.78 ±0.25^{b}	59.97 ±0.62^{b}	37.96 ±0.18^{d}	35.54 ±0.27^{d}	33.89 ±0.43^{d}
Kaempferol 3-O-p-coumaroylhexoside isomer	39.74 ±0.26^{a}	33.69 ±0.27^{b}	31.78 ±0.69^{c}	31.59 ±0.28^{c}	31.43 ±0.41^{c}	30.99 ±0.68^{c}	30.67 ±0.47^{c}	30.01 ±0.30^{c}
Quercetin-3-O-glucosylrutinoside	105.78 ±1.23^{a}	103.67 ±1.09^{ab}	102.15 ±1.11^{b}	101.56 ±1.32^{b}	95.94 ±1.06^{c}	91.33 ±1.47^{c}	85.88 ±0.99^{d}	84.99 ±1.52^{d}
Quercetin 3-O-dirhamnosylhexoside	21.90 ±0.39^{a}	20.30 ±0.48^{ab}	20.25 ±0.28^{ab}	20.10 ±0.34^{ab}	19.88 ±0.51^{b}	19.55 ±0.47^{bc}	19.46 ±0.71^{c}	19.39 ±0.21^{c}
Quercetin hexoside-deoxyhexoside	90.25 ±1.33^{a}	85.12 ±1.29^{b}	81.78 ±1.62^{c}	80.78 ±1.17^{c}	74.99 ±1.44^{d}	73.14 ±1.62^{e}	70.20 ±1.87^{f}	70.21 ±1.09^{f}
Quercetin 3-O-glucoside	40.00 ±1.00^{a}	38.14 ±1.32^{b}	36.67 ±1.06^{c}	35.37 ±1.28^{cd}	33.98 ±1.45^{d}	32.65 ±1.02^{de}	31.34 ±1.09^{e}	27.23 ±1.30^{f}
Quercetin 3-O-galactoside	30.67 ±1.09^{a}	29.32 ±1.12^{a}	26.56 ±0.98^{b}	25.72 ±0.65^{b}	23.69 ±1.00^{c}	22.11 ±0.97^{c}	20.49 ±0.68^{d}	18.03 ±0.24^{e}
Kaempferol 3-O-p-glucosylrutinoside isomer	36.70 ±1.30^{b}	35.67 ±0.99^{cd}	36.09 ±1.06^{bc}	35.20 ±0.94^{d}	34.44 ±0.58^{e}	35.89 ±1.21^{c}	37.14 ±1.36^{b}	40.87 ±1.08^{a}
Kaempferol 3-O-2",6"-di-p-coumaroylglucoside	17.30 ±0.12^{a}	16.10 ±0.09^{c}	16.30 ±0.11^{bc}	16.32 ±0.10^{bc}	16.36 ±0.06^{b}	16.38 ±0.09^{b}	16.49 ±0.11^{b}	16.57 ±0.08^{b}
Kaempferol 3-O-rutinoside isomer	29.00 ±1.06^{a}	25.32 ±1.22^{b}	25.00 ±0.68^{b}	24.56 ±1.03^{bc}	24.03 ±0.89^{bc}	23.45 ±0.65^{c}	23.01 ±1.21^{cd}	22.59 ±1.05^{d}
Kaempferol 3-O-glucoside	21.16 ±0.85^{a}	21.01 ±1.06^{a}	20.88 ±0.16^{ab}	20.68 ±1.22^{b}	20.02 ±0.36^{c}	19.81 ±0.24^{cd}	19.38 ±0.56^{d}	18.90 ±0.48^{e}
Kaempferol 3-O-galactoside	27.25 ±0.58^{a}	25.44 ±0.24^{b}	25.01 ±0.36^{bc}	24.63 ±0.59^{c}	24.48 ±0.14^{c}	24.36 ±0.51^{cd}	24.22 ±0.26^{d}	24.06 ±0.54^{d}
Quercetin 3-O-acylglycoside isomer	65.45 ±1.25^{a}	64.35 ±0.98^{a}	61.78 ±1.06^{b}	61.33 ±1.02^{b}	60.34 ±0.87^{c}	60.10 ±1.12^{c}	59.71 ±0.58^{c}	59.38 ±1.10^{cd}
Kaempferol 3-O-p-coumaroylrhamnosyldihexoside isomer	46.56 ±1.23^{a}	46.43 ±1.45^{a}	46.04 ±1.36^{ab}	45.57 ±1.09^{b}	45.31 ±1.51^{bc}	45.08 ±1.13^{c}	45.00 ±1.08^{c}	45.00 ±0.77^{c}
Quercetin 3-O-p-coumaroyldirhamnosylhexoside isomer	50.12 ±1.66^{a}	48.09 ±1.58^{de}	49.67 ±1.32^{ab}	49.01 ±1.54^{bc}	48.66 ±1.06^{c}	48.41 ±1.24^{c}	48.28 ±1.07^{cd}	47.01 ±1.10^{e}
Kaempferol 3-O-p-coumaroylrhamnosyldihexoside isomer	21.08 ±0.36^{a}	17.97 ±0.21^{b}	17.13 ±0.25^{bc}	16.88 ±0.41^{c}	16.75 ±0.31^{c}	16.65 ±0.09^{cd}	16.57 ±0.12^{d}	15.94 ±0.11^{e}
Kaempferol 3-O-p-coumaroylrhamnosyldihexoside isomer	25.45 ±0.32^{a}	20.21 ±0.15^{f}	24.13 ±0.38^{b}	23.98 ±0.41^{b}	23.05 ±0.21^{c}	22.89 ±0.15^{cd}	22.56 ±0.16^{d}	21.14 ±0.08^{e}
Isorhamnetin-acylated-hexoside	0.85 ±0.02^{a}	0.67 ±0.03^{cd}	0.78 ±0.01^{ab}	0.71 ±0.02^{bc}	0.65 ±0.02^{cd}	0.59 ±0.01^{d}	0.52 ±0.01^{de}	0.48 ±0.01^{e}
Isorhamnetin-acylated-hexoside	2.67 ±0.12^{a}	2.55 ±0.13^{b}	2.53 ±0.09^{b}	2.43 ±0.10^{c}	2.39 ±0.15^{c}	2.31 ±0.11^{d}	2.15 ±0.08^{e}	2.03 ±0.07^{f}
SUM	751.33 ±25.34^{a}	683.65 ±24.54^{b}	687.23 ±22.67^{b}	679.25 ±23.94^{bc}	658.12 ±22.03^{c}	624.63 ±22.50^{d}	609.21 ±22.01^{e}	598.27 ±20.06^{e}
**Purine Alkaloids**								
Theanine	2.12 ±0.15^{e}	2.01 ±0.12^{f}	2.23 ±0.03^{d}	2.27 ±0.14^{cd}	2.30 ±0.09^{bc}	2.35 ±0.05^{b}	2.65 ±0.1^{va}	2.66 ±0.11^{a}
Theobromine	40.46 ±1.03^{cd}	29.69 ±0.98^{f}	34.36 ±0.85^{e}	36.94 ±0.67^{de}	40.21 ±1.01^{cd}	42.10 ±0.77^{c}	48.4 ±0.98^{b}	63.18 ±1.10^{a}
SUM	42.58 ±2.04^{c}	31.70 ±1.91^{e}	36.59 ±1.52^{d}	39.21 ±1.40^{cd}	42.51 ±1.91^{c}	44.45 ±1.42^{c}	51.05 ±1.87^{b}	65.84 ±2.10^{a}
**Theaflavins**								
Theaflavin isomer	40.30 ±1.10^{a}	36.78 ±1.03^{cd}	40.21 ±0.98^{a}	40.09 ±1.21^{a}	38.32 ±1.04^{b}	37.56 ±1.14^{bc}	35.69 ±0.66^{de}	34.89 ±0.99^{e}
Theaflavin isomer	10.24 ±0.51^{a}	9.80 ±0.26^{bc}	10.12 ±0.31^{ab}	9.90 ±0.48^{b}	9.45 ±0.51^{c}	8.10 ±0.28^{d}	7.89 ±0.17^{de}	7.65 ±0.61^{e}
SUM	50.54 ±2.79^{a}	46.58 ±2.23^{bc}	50.33 ±2.23^{a}	49.99 ±2.93^{a}	47.77 ±2.68^{b}	45.66 ±2.46^{c}	43.58 ±1.44^{cd}	42.54 ±2.77^{d}
**Flavones**								
Apigenin C-hexoside-C-pentoside	40.34 ±1.78^{a}	38.89 ±1.89^{b}	38.90 ±1.70^{b}	38.83 ±1.82^{b}	38.67 ±1.51^{bc}	38.45 ±1.52^{c}	38.40 ±1.75^{c}	38.23 ±1.71^{c}
TOTAL	4414.39 ±108.25^{b}	3279.62 ±111.58^{h}	3531.63 ±93.72^{g}	3705.98 ±103.32^{f}	3855.61 ±102.45^{e}	3973.99 ±112.05^{d}	4027.36 ±110.70^{c}	4738.68 ±107.01^{a}

Means of three separate analyses ± standard deviation. Duncan's test reveals significant differences ($p < 0.05$) between values in the same rows with different letters (a-v).

Table S4. The content of phenolic compounds (mg/100 mL) in kombucha 3 (K3) during fermentation

Compound	Day 0	Day 2	Day 4	Day 6	Day 8	Day 10	Day 12	Day 14
**Phenolic acids and derivatives**								
Quinic acid	2.31 ±0.13^{a}	2.00 ±0.14^{b}	2.20 ±0.09^{a}	2.03 ±0.15^{b}	2.00 ±0.09^{b}	1.96 ±0.06b^{c}	1.90 ±0.08^{cd}	1.85 ±0.10^{d}
Caffeoylhexoside	1.12 ±0.01^{c}	1.00 ±0.01^{c}	1.05 ±0.02^{c}	1.58 ±0.02^{b}	1.59 ±0.08^{b}	1.62 ±0.07^{ab}	1.62 ±0.02^{ab}	1.65 ±0.03^{a}
Galloylhexose isomer	0.15 ±0.01^{a}	0.11 ±0.01^{ab}	0.14 ±0.01^{a}	0.09 ±0.00^{b}	0.02 ±0.00^{c}	0.00 ±0.00^{d}	0.00 ±0.00^{d}	0.00 ±0.00^{d}
Theogallin	8.25 ±0.23^{bc}	8.00 ±0.15^{e}	8.09 ±0.21^{d}	8.19 ±0.18^{c}	8.25 ±0.09^{bc}	8.34 ±0.14^{ab}	8.36 ±0.15^{ab}	8.41 ±0.23^{a}
Galloylhexose isomer	26.75 ±0.36^{a}	24.74 ±0.28^{b}	20.02 ±0.12^{c}	19.88 ±0.32^{cd}	18.86 ±0.21^{d}	18.15 ±0.17^{d}	12.33 ±0.18^{e}	10.05 ±0.10^{f}
Theogallin dimer	80.33 ±1.23^{a}	67.49 ±1.52^{d}	76.16 ±1.18^{b}	74.67 ±1.09^{c}	74.65 ±1.21^{c}	75.01 ±1.09^{c}	75.89 ±1.11^{bc}	75.99 ±1.33^{b}
Coffeoylhexose	20.95 ±0.89^{a}	15.56 ±1.03^{f}	20.44 ±0.65^{ab}	20.09 ±0.47^{bc}	19.87 ±0.35^{c}	19.05 ±0.84^{d}	18.92 ±0.15^{d}	18.03 ±0.29^{e}
Caffeoylhexoside	3.78 ±0.12^{a}	3.45 ±0.09^{b}	3.01 ±0.14^{c}	2.99 ±0.15^{cd}	2.86 ±0.06d	2.56 ±0.30^{e}	2.44 ±0.47^{f}	2.32 ±0.11^{f}
3-galloylquinic acid	1.40 ±0.03^{a}	0.87 ±0.02^{e}	1.30 ±0.03^{b}	1.26 ±0.01^{bc}	1.22 ±0.02^{c}	1.09 ±0.01^{d}	0.84 ±0.02^{e}	0.67 ±0.03^{f}
cis-3-Caffeoylquinic acid	17.25 ±0.58^{a}	13.34 ±0.36^{f}	12.43 ±0.45^{g}	14.01 ±0.29^{e}	14.30 ±0.87^{de}	14.77 ±0.25^{c}	14.99 ±0.19^{bc}	15.30 ±0.61^{b}
5-Caffeoylquinic acid	15.22 ±0.09^{c}	14.01 ±0.16^{d}	14.99 ±0.08^{c}	15.21 ±0.02^{c}	15.28 ±0.14^{bc}	15.34 ±0.10^{ab}	15.56 ±0.06^{a}	15.53 ±0.15^{a}
cis-4-p-Coumaroylquinic acid	60.60 ±0.58^{a}	40.44 ±0.39^{d}	48.09 ±0.27^{c}	48.17 ±0.45c	50.85 ±0.88^{c}	55.05 ±1.09^{b}	59.17 ±0.58^{a}	60.67 ±0.68^{a}
Galloylhexose isomer	2.47 ±0.16^{a}	1.02 ±0.09^{bc}	1.15 ±0.05^{b}	1.09 ±0.01^{b}	0.97 ±0.02^{cd}	0.89 ±0.03^{d}	0.76 ±0.02^{e}	0.65 ±0.01^{f}
Caffeoyl-N-tryptophanrhamnoside	2.89 ±0.1^{e}	2.99 ±0.06^{de}	3.19 ±0.09^{d}	3.50 ±0.10^{c}	3.65 ±0.09^{bc}	3.71 ±0.06^{b}	3.73 ±0.03^{ab}	3.76 ±0.00^{a}
5-p-Coumaroylquinic acid	52.48 ±0.65^{a}	45.16 ±0.28^{b}	45.34 ±0.51^{b}	44.88 ±0.39^{b}	44.53 ±0.48^{b}	44.77 ±0.57^{b}	44.51 ±0.24^{b}	44.73 ±0.36^{b}
3-p-Coumaroylquinic acid	995.95 ±12.95^{c}	920.26 ±20.36^{f}	961.54 ±14.78^{e}	970.81 ±22.02^{d}	993.34 ±18.18^{c}	1006.51 ±21.36^{b}	1012.73 ±20.18^{b}	1239.87 ±14.06^{a}
Gallic acid	30.95 ±1.03^{bc}	26.65 ±0.98^{f}	26.46 ±1.2^{f}	27.56 ±0.04^{e}	28.72 ±1.03^{d}	30.02 ±0.85^{c}	31.46 ±0.22^{b}	35.47 ±1.00^{a}
Trigalloylhexose	10.62 ±0.16^{a}	10.26 ±0.21^{b}	9.49 ±0.08^{d}	9.65 ±0.12^{c}	9.53 ±0.06^{cd}	9.48 ±0.10^{d}	3.12 ±0.08^{e}	2.56 ±0.03^{e}
SUM	1333.47 ±33.45^{b}	1197.35 ±45.28^{e}	1255.09 ±34.57^{d}	1265.14 ±44.74^{cd}	1289.96 ±41.33^{c}	1308.32 ±46.92^{bc}	1308.33 ±41.19^{bc}	1537.51 ±33.12^{a}
**Flavan-3-ols and Proanthocyanidins**								
(+)-Gallocatechin	11.28 ±0.63^{c}	8.00 ±0.28^{e}	10.87 ±0.54^{d}	11.04 ±0.72^{cd}	11.21 ±0.92^{c}	11.67 ±0.29^{bc}	12.07 ±0.15^{b}	13.32 ±0.41^{a}
Gallocatechin dimer unknown isomer	1.00 ±0.09^{c}	0.87 ±0.06^{d}	1.01 ±0.08^{c}	1.06 ±0.05^{c}	1.11 ±0.06^{bc}	1.40 ±0.03^{a}	1.15 ±0.04^{b}	1.19 ±0.01^{b}
Gallocatechin dimer unknown isomer	1.60 ±0.02^{a}	1.09 ±0.02^{b}	0.87 ±0.01^{d}	0.89 ±0.02^{d}	0.92 ±0.02^{cd}	0.93 ±0.00^{cd}	0.96 ±0.00^{c}	0.99 ±0.01^{bc}
(+)-Gallocatechin dimer	46.78 ±1.09^{a}	34.44 ±0.86^{f}	35.99 ±1.12^{e}	36.04 ±0.68^{e}	36.86 ±1.06^{de}	37.30 ±1.11^{d}	39.56 ±0.99^{c}	42.54 ±0.85^{b}
(-)-Epigallocatechin	3.77 ±0.12^{d}	3.49 ±0.08^{e}	3.53 ±0.15^{e}	3.91 ±0.06^{c}	3.95 ±0.13^{c}	4.31 ±0.10^{b}	4.33 ±0.11^{b}	4.42 ±0.05^{a}
(+)-Catechin	1987.44 ±12.36^{b}	1789.33 ±14.89^{e}	1821.72 ±20.54^{c}	1896.35 ±18.22^{d}	1939.91 ±21.2^{c}	1937.90 ±16.37^{c}	1989.61 ±10.8^{b}	2135.76 ±24.15^{a}
B-type procyanidin dimer	25.62 ±0.32^{a}	19.19 ±1.15^{b}	18.77 ±0.58^{b}	15.37 ±0.22^{c}	14.90 ±1.06^{c}	13.67 ±0.14^{d}	12.54 ±0.06^{e}	11.33 ±0.36^{f}
(-)-Methylepigallocatechin gallate	30.05 ±0.15^{a}	25.94 ±0.25^{e}	29.15 ±0.36^{b}	28.98 ±0.24^{b}	28.87 ±0.51^{c}	28.76 ±0.29^{c}	27.39 ±0.14^{d}	26.90 ±0.30^{d}
B-type procyanidin dimer	35.37 ±0.51^{a}	34.30 ±0.32^{b}	32.58 ±0.84^{d}	33.77 ±0.19^{c}	31.86 ±0.14^{e}	30.32 ±0.10^{f}	29.19 ±0.16^{g}	28.79 ±0.21^{h}
Procyanidin trimer isomer	17.66 ±0.14^{a}	13.48 ±0.08^{b}	12.95 ±0.10^{bc}	12.78 ±0.22^{cd}	12.12 ±0.16^{d}	12.01 ±0.13^{d}	11.43 ±0.08^{e}	11.09 ±0.09^{e}
(-)-Epicatechin	9.35 ±0.10^{a}	6.42 ±0.03^{e}	7.17 ±0.12^{d}	7.24 ±0.08^{cd}	7.40 ±0.15^{c}	7.73 ±0.06^{b}	7.89 ±0.23^{b}	7.93 ±0.07^{b}
(-)-Epigallocatechin gallate	117.75 ±1.22^{a}	114.40 ±2.13^{c}	104.84 ±1.09^{f}	106.42 ±3.56^{e}	109.50 ±2.08^{d}	112.57 ±1.48^{c}	116.67 ±1.58^{b}	117.93 ±0.89^{a}
Gallocatechin gallate isomer	3.29 ±0.31^{a}	3.03 ±0.21^{b}	2.97 ±0.08^{b}	2.68 ±0.12^{c}	2.56 ±0.20^{d}	2.40 ±0.15^{e}	2.33 ±0.16^{f}	2.21 ±0.09^{g}
B-type procyanidin dimer	40.25 ±0.25^{ab}	39.26 ±0.34^{c}	40.97 ±0.52^{a}	40.56 ±0.18^{ab}	40.26 ±0.02^{ab}	39.95 ±0.51^{b}	39.61 ±0.10^{b}	39.40 ±0.08^{bc}
(+)-Epicatechin gallate	160.43 ±1.63^{e}	150.82 ±1.22^{f}	160.15 ±2.15^{e}	168.77 ±4.80^{d}	169.45 ±1.09^{d}	190.34 ±1.32^{c}	200.36 ±2.54^{b}	205.98 ±2.09^{a}

Gallocatechin unknown derivative	0.00 ±0^{c}	0.00 ±0.00^{c}	0.00 ±0.00^{c}	0.00 ±0.00^{c}	0.00 ±0.00^{c}	0.00 ±0.00^{c}	0.29 ±0.01^{b}	0.66 ±0.01^{a}
SUM	2491.64 ±32.81^{b}	2244.06 ±37.97^{f}	2283.54 ±48.98^{e}	2365.86 ±50.85^{d}	2410.88 ±49.88^{c}	2430.36 ±38.24^{c}	2495.38 ±29.70^{b}	2650.44 ±51.39^{a}
**Flavonols**								
Mirycetin glucoside	0.76 ±0.02^{a}	0.68 ±0.02^{b}	0.52 ±0.01^{c}	0.30 ±0.01^{d}	0.07 ±0.00^{e}	0.00 ±0.00^{f}	0.00 ±0.00^{f}	0.00 ±0.00^{f}
Mirycetin galactoside	2.56 ±0.12^{a}	2.14 ±0.08^{b}	1.67 ±0.10^{c}	1.24 ±0.06^{d}	0.67 ±0.30^{e}	0.55 ±0.02^{e}	0.43 ±0.08^{ef}	0.38 ±0.01^{f}
Quercetin-3-O-glucosylrutinoside	90.68 ±1.32^{a}	84.18 ±0.69^{b}	89.77 ±1.25^{a}	89.89 ±0.84^{a}	78.65 ±0.58^{c}	63.77 ±1.22^{d}	50.50 ±0.99^{e}	48.71 ±1.35^{e}
Kaempferol 3-O-p-coumaroylhexoside isomer	49.70 ±1.09^{a}	48.74 ±0.58^{ab}	47.54 ±1.21^{bc}	46.68 ±1.03^{cd}	45.77 ±0.87^{d}	45.24 ±1.41^{d}	45.67 ±0.65^{d}	45.50 ±0.87^{d}
Quercetin-3-O-glucosylrutinoside	130.64 ±2.15^{a}	130.22 ±1.98^{a}	127.98 ±2.54^{b}	125.49 ±0.30^{c}	123.71 ±2.14^{d}	120.02 ±3.57^{e}	118.99 ±1.65^{f}	117.72 ±2.01^{f}
Quercetin 3-O-dirhamnosylhexoside	27.18 ±0.09^{a}	25.34 ±0.13^{b}	25.29 ±0.08^{b}	25.11 ±0.06^{bc}	24.86 ±0.11^{c}	24.48 ±0.09^{c}	24.37 ±0.13^{c}	24.29 ±0.07^{c}
Quercetin hexoside-deoxyhexoside	123.78 ±2.99^{a}	117.88 ±1.56^{b}	114.04 ±3.12^{bc}	112.98 ±4.55^{c}	106.23 ±2.22^{d}	104.11 ±1.09^{de}	100.31 ±1.46^{e}	99.74 ±1.82^{e}
Quercetin 3-O-glucoside	54.67 ±2.03^{a}	53.86 ±1.65^{ab}	52.17 ±2.08^{bc}	51.67 ±1.44^{cd}	50.07 ±1.06^{d}	47.89 ±1.28^{e}	46.04 ±1.65^{ef}	45.45 ±0.08^{f}
Quercetin 3-O-galactoside	40.27 ±1.66^{a}	37.71 ±1.05^{b}	35.64 ±1.13^{c}	33.29 ±1.48^{d}	30.10 ±1.03^{e}	29.89 ±0.89^{e}	25.22 ±1.10^{f}	23.31 ±1.08^{g}
Kaempferol 3-O-p-glucosylrutinoside isomer	44.20 ±1.51^{a}	40.22 ±2.01^{b}	41.30 ±0.85^{b}	41.01 ±1.22^{b}	40.68 ±1.06^{b}	40.90 ±0.47^{b}	41.01 ±0.65^{b}	41.09 ±1.30^{b}
Kaempferol 3-O-2",6"-di-p-coumaroylglucoside	18.99 ±0.23^{a}	18.50 ±0.21^{ef}	18.46 ±0.18^{f}	18.50 ±0.08^{ef}	18.57 ±0.12^{de}	18.61 ±0.10^{cd}	18.68 ±0.14^{bc}	16.72 ±0.21^{ab}
Kaempferol 3-O-rutinoside isomer	35.35 ±0.32^{a}	33.18 ±0.25^{ab}	32.98 ±0.62^{b}	32.04 ±0.12^{bc}	31.76 ±0.08^{cd}	31.09 ±0.24^{d}	30.30 ±0.04^{e}	29.41 ±0.01^{f}
Kaempferol 3-O-glucoside	30.33 ±0.21^{a}	29.15 ±0.21^{ab}	27.87 ±0.06^{bc}	26.27 ±0.3^{cd}	25.02 ±0.11^{de}	24.78 ±0.14^{ef}	24.28 ±0.51^{ef}	23.78 ±0.01^{f}
Kaempferol 3-O-galactoside	35.61 ±0.21^{a}	32.18 ±0.25^{bc}	31.63 ±0.06^{c}	31.60 ±0.04^{c}	32.84 ±0.12^{b}	32.14 ±0.31^{bc}	31.80 ±0.25^{c}	31.78 ±0.10^{c}
Quercetin 3-O-acylglycoside isomer	69.72 ±1.13^{a}	68.65 ±1.22^{ab}	68.43 ±0.58^{ab}	68.10 ±0.06^{b}	67.59 ±1.34^{bc}	67.44 ±0.15^{bc}	67.03 ±1.20^{c}	65.65 ±1.33^{d}
Kaempferol 3-O-p-coumaroylrhamnosyldihexoside isomer	50.33 ±1.2^{a}	50.00 ±0.69^{ab}	49.77 ±0.51^{b}	49.34 ±1.32^{b}	49.09 ±1.52^{bc}	49.00 ±1.06^{bc}	48.66 ±0.54^{c}	48.51 ±1.37^{c}
Quercetin 3-O-p-coumaroyldirhamnosylhexoside isomer	50.20 ±0.89^{a}	49.50 ±1.2^{ab}	50.00 ±1.33^{a}	49.97 ±2.02^{a}	49.67 ±1.65^{ab}	49.44 ±1.84^{bc}	49.20 ±2.01^{bc}	49.11 ±1.99^{c}
Kaempferol 3-O-p-coumaroylrhamnosyldihexoside isomer	22.54 ±0.41^{a}	22.01 ±0.03^{b}	21.67 ±0.15^{c}	21.15 ±0.20^{d}	21.00 ±0.16^{de}	20.81 ±0.02^{e}	20.80 ±0.14^{e}	20.67 ±0.01^{f}
Kaempferol 3-O-p-coumaroylrhamnosyldihexoside isomer	25.70 ±0.01^{a}	21.22 ±0.21^{d}	24.76 ±0.13^{b}	24.56 ±0.15^{b}	24.20 ±0.17^{b}	24.15 ±0.16^{b}	24.03 ±0.08^{bc}	23.99 ±0.12^{c}
Isorhamnetin-acylated-hexoside	0.99 ±0.02^{a}	0.97 ±0.03^{a}	0.90 ±0.02^{b}	0.87 ±0.01^{b}	0.86 ±0.00^{b}	0.76 ±0.01^{c}	0.69 ±0.01^{d}	0.64 ±0.00^{d}
Isorhamnetin-acylated-hexoside	2.90 ±0.10^{a}	2.78 ±0.05^{b}	2.65 ±0.03^{c}	2.63 ±0.08^{c}	2.57 ±0.01^{cd}	2.51 ±0.02^{d}	2.44 ±0.00^{e}	2.40 ±0.02^{e}
SUM	907.10 ±30.67^{a}	869.11 ±24.42^{b}	865.04 ±27.78^{b}	852.69 ±26.62^{bc}	823.98 ±25.37^{c}	797.58 ±24.40^{d}	770.45 ±23.00^{de}	758.85 ±23.83^{e}
**Purine Alkaloids**								
Theanine	1.80 ±0.06^{a}	1.50 ±0.10^{e}	1.65 ±0.08^{cd}	1.60 ±0.03^{d}	1.66 ±0.07^{c}	1.70 ±0.10^{bc}	1.72 ±0.09^{b}	1.75 ±0.03^{b}
Theobromine	35.15 ±0.15^{b}	28.78 ±0.20^{g}	30.33 ±0.14^{f}	31.03 ±0.19^{e}	32.16 ±0.10^{d}	32.65 ±0.09^{cd}	33.21 ±0.15^{c}	39.98 ±0.07^{a}
SUM	36.95 ±0.36^{b}	30.28 ±0.52^{f}	31.98 ±0.38^{e}	32.63 ±0.38^{de}	33.82 ±0.29^{d}	34.35 ±0.33^{cd}	34.93 ±0.42^{c}	41.73 ±0.17^{a}
**Theaflavins**								
Theaflavin isomer	30.24 ±0.20^{a}	25.42 ±0.32^{c}	26.16 ±0.15^{b}	26.07 ±0.16^{b}	25.65 ±0.21^{c}	25.04 ±0.20^{cd}	24.55 ±0.08^{d}	23.91 ±0.15^{e}
Theaflavin isomer	7.19 ±0.08^{a}	6.84 ±0.10^{b}	7.09 ±0.09^{ab}	6.89 ±0.12^{b}	6.66 ±0.20^{c}	6.56 ±0.13^{cd}	6.49 ±0.10^{d}	6.33 ±0.22^{e}
SUM	37.43 ±0.48^{a}	32.26 ±0.73^{c}	33.25 ±0.42^{b}	32.96 ±0.48^{bc}	32.31 ±0.71^{c}	31.60 ±0.57^{de}	31.04 ±0.31^{e}	30.24 ±0.64^{f}
**Flavones**								
Apigenin C-hexoside-C-pentoside	52.35 ±2.17^{a}	50.89 ±3.74^{b}	50.84 ±1.89^{b}	50.67 ±3.55^{b}	50.64 ±2.30^{b}	50.5 ±3.48^{b}	50.44 ±3.67^{b}	50.40 ±2.20^{b}
TOTAL	4858.94 ±99.94^{b}	4423.95 ±112.65^{g}	4519.74 ±114.02^{f}	4599.95 ±126.63^{e}	4641.589 ±119.89^{d}	4652.71 ±113.95^{cd}	4690.57 ±98.29^{c}	5069.17 ±111.35^{a}

Means of three separate analyses ± standard deviation. Duncan's test reveals significant differences ($p < 0.05$) between values in the same rows with different letters (a-g).

Table S5. The content of phenolic compounds (mg/100 mL) in kombucha 4 (K4) during fermentation

Compound	Day 0	Day 2	Day 4	Day 6	Day 8	Day 10	Day 12	Day 14
**Phenolic acids and derivatives**								
Quinic acid	2.66 ±0.13^{a}	2.50 ±0.08^{b}	2.61 ±0.15^{a}	2.54 ±0.06^{b}	2.48 ±0.12^{bc}	2.43 ±0.10^{cd}	2.37 ±0.05^{d}	2.35 ±0.13^{d}
Caffeoylhexoside	1.45 ±0.08^{bc}	1.39 ±0.05^{c}	1.40 ±0.10^{c}	1.45 ±0.04^{bc}	1.46 ±0.03^{bc}	1.52 ±0.06^{b}	1.60 ±0.05^{a}	1.61 ±0.02^{a}
Galloylhexose isomer	0.10 ±0.01^{a}	0.03 ±0.00^{b}	0.00 ±0.00^{c}	0.00 ±0.00^{c}	0.00 ±0.00^{c}	0.00 ±0.00^{c}	0.00 ±0.00^{c}	0.00 ±0.00^{c}
Caffeoylhexoside	5.05 ±0.21^{cd}	4.98 ±0.13^{d}	4.99 ±0.08^{d}	5.05 ±0.14^{cd}	5.12 ±0.10^{c}	5.20 ±0.21^{b}	5.22 ±0.06^{ab}	5.29 ±0.03^{a}
Theogallin	19.21 ±0.36^{a}	17.31 ±0.25^{b}	16.65 ±0.31^{c}	15.99 ±0.48^{d}	15.03 ±0.27^{e}	14.21 ±0.32^{f}	13.55 ±0.08^{g}	12.81 ±0.41^{h}
Galloylhexose isomer	61.34 ±1.51^{a}	55.17 ±0.22^{d}	59.93 ±1.32^{ab}	59.13 ±0.58^{ab}	58.84 ±0.99^{bc}	57.66 ±1.03^{bc}	57.12 ±0.65^{bc}	56.99 ±1.24^{c}
Theogallin dimer	27.98 ±0.69^{a}	25.43 ±0.58^{b}	25.33 ±0.34^{b}	25.14 ±0.95^{bc}	24.97 ±0.12^{c}	24.67 ±0.09^{cd}	24.54 ±0.51^{d}	24.39 ±0.28^{d}
Coffeoylhexose	3.88 ±0.12^{a}	3.60 ±0.05^{bc}	3.67 ±0.17^{b}	3.60 ±0.12^{bc}	3.54 ±0.10^{c}	3.45 ±0.09^{d}	3.32 ±0.11^{d}	3.30 ±0.05^{d}
Caffeoylhexoside	0.82 ±0.05^{a}	0.55 ±0.02^{b}	0.59 ±0.01^{b}	0.45 ±0.00^{c}	0.32 ±0.01^{d}	0.30 ±0.01^{d}	0.21 ±0.00^{e}	0.09 ±0.00^{f}
3-galloylquinic acid	25.15 ±0.13^{b}	24.56 ±0.32^{c}	23.45 ±0.25^{e}	24.01 ±0.14^{d}	24.55 ±0.20^{cd}	24.67 ±0.06^{c}	24.92 ±0.15^{bc}	25.63 ±0.21^{a}
cis-3-Caffeoylquinic acid	22.98 ±0.21^{a}	21.15 ±0.36^{d}	20.11 ±0.25^{e}	21.05 ±0.15^{d}	21.78 ±0.06^{c}	21.89 ±0.41^{c}	22.31 ±0.25^{b}	22.46 ±0.31^{b}
5-Caffeoylquinic acid	0.77 ±0.03^{c}	0.65 ±0.02^{d}	0.82 ±0.03^{b}	0.83 ±0.01^{b}	0.85 ±0.04^{b}	0.90 ±0.01^{ab}	0.93 ±0.02^{a}	0.94 ±0.01^{a}
Caffeoyl-N-tryptophan	19.78 ±0.16^{a}	18.02 ±0.32^{f}	18.55 ±0.25^{e}	18.76 ±0.27^{de}	18.88 ±0.14^{d}	19.01 ±0.51^{c}	19.35 ±0.26^{b}	19.40 ±0.11^{b}
cis-4-p-Coumaroylquinic acid	3.01 ±0.08^{b}	2.87 ±0.15^{c}	2.82 ±0.09^{c}	2.89 ±0.13^{c}	2.05 ±0.14^{d}	3.01 ±0.05^{b}	3.17 ±0.04^{ab}	3.24 ±0.10^{a}
Caffeoylquinic acid	82.15 ±1.32^{b}	76.10 ±0.69^{e}	80.24 ±1.52^{d}	81.53 ±2.15^{c}	81.88 ±0.68^{c}	82.12 ±1.95^{b}	82.34 ±2.01^{ab}	82.59 ±2.44^{a}
Caffeoyl-N-tryptophanrhamnoside	33.04 ±0.25^{a}	30.33 ±0.31^{b}	29.77 ±0.16^{b}	27.15 ±0.51^{c}	27.01 ±0.21^{c}	24.15 ±0.14^{d}	20.01 ±0.33^{e}	18.46 ±0.15^{f}
5-p-Coumaroylquinic acid	7.15 ±0.19^{a}	6.01 ±0.20^{e}	5.97 ±0.15^{e}	6.01 ±0.13^{e}	6.11 ±0.22^{d}	6.18 ±0.14^{c}	6.22 ±0.10^{bc}	6.25 ±0.05^{b}
3-p-Coumaroylquinic acid	0.40 ±0.02^{a}	0.32 ±0.01^{b}	0.17 ±0.01^{c}	0.03 ±0.00^{d}	0.00 ±0.00^{e}	0.00 ±0.00^{e}	0.00 ±0.00^{e}	0.00 ±0.00^{e}
Gallic acid	2.54 ±0.01^{a}	2.49 ±0.12^{ab}	2.40 ±0.06^{bc}	2.35 ±0.03^{cd}	2.29 ±0.00^{d}	2.07 ±0.05^{e}	1.94 ±0.09^{ef}	1.87 ±0.12^{f}
Trigalloylhexose	27.15 ±0.52^{a}	24.34 ±0.32^{b}	24.03 ±0.09^{bc}	23.95 ±0.12^{c}	23.68 ±0.21^{cd}	23.52 ±0.41^{d}	23.12 ±0.03^{de}	22.93 ±0.15^{e}
SUM	346.61 ±10.53^{a}	317.80 ±7.27^{c}	323.50 ±9.25^{b}	321.91 ±10.41^{b}	320.84 ±6.30^{b}	316.96 ±9.77^{c}	312.24 ±8.30^{cd}	310.60 ±10.06^{d}
**Flavan-3-ols and Proanthocyanidins**								
(+)-Gallocatechin dimer	0.87 ±0.02^{a}	0.66 ±0.02^{b}	0.65 ±0.01^{b}	0.58 ±0.01^{c}	0.53 ±0.02^{c}	0.00 ±0.00^{d}	0.00 ±0.00^{d}	0.00 ±0.00^{d}
(-)-Epigallocatechin	40.85 ±0.36^{a}	28.20 ±0.28^{d}	28.99 ±0.65^{cd}	29.12 ±0.26^{c}	29.22 ±0.14^{bc}	29.97 ±0.28^{b}	30.13 ±0.41^{b}	30.65 ±0.36^{b}
(+)-Catechin	3.50 ±0.15^{a}	3.22 ±0.23^{b}	3.18 ±0.41^{b}	3.11 ±0.3^{bc}	3.05 ±0.11^{c}	2.99 ±0.09^{cd}	2.56 ±0.11^{d}	2.01 ±0.08^{e}
B-type procyanidin dimer	2658.66 ±6.15^{c}	2603.37 ±2.22^{e}	2645.76 ±0.99^{d}	2664.33 ±5.24^{c}	2678.19 ±1.68^{bc}	2688.02 ±4.13^{b}	2693.98 ±3.58^{ab}	2699.99 ±0.99^{a}
(-)-Methylepigallocatechin gallate	1.66 ±0.01^{e}	1.68 ±0.10^{e}	1.73 ±0.09^{de}	1.77 ±0.13^{d}	1.83 ±0.05^{c}	1.90 ±0.03^{b}	1.94 ±0.08^{ab}	1.99 ±0.09^{a}
B-type procyanidin dimer	17.36 ±0.62^{b}	12.71 ±0.09^{d}	18.66 ±0.13^{a}	16.30 ±0.22^{cd}	16.68 ±0.41^{c}	10.35 ±0.26^{f}	11.57 ±0.31^{e}	12.08 ±0.25^{e}
B-type procyanidin dimer	44.89 ±0.54^{a}	44.56 ±0.26^{b}	41.90 ±0.32^{c}	41.77 ±0.30^{c}	40.35 ±0.28^{d}	38.76 ±0.41^{e}	38.01 ±0.16^{ef}	37.67 ±0.08^{f}
B-type procyanidin dimer	25.15 ±0.13^{a}	23.44 ±0.25^{b}	22.78 ±0.62^{bc}	22.03 ±0.32^{c}	21.56 ±0.23^{cd}	20.99 ±0.15^{d}	19.76 ±0.25^{e}	19.00 ±0.16^{f}
Procyanidin trimer isomer	71.18 ±1.55^{a}	65.55 ±0.69^{d}	65.60 ±1.12^{cd}	65.88 ±0.58^{c}	65.96 ±0.13^{c}	66.01 ±1.21^{bc}	66.10 ±2.22^{b}	66.13 ±1.03^{b}
(-)-Epicatechin	90.34 ±2.65^{d}	90.01 ±1.23^{d}	91.12 ±3.21^{cd}	91.66 ±1.09^{c}	91.89 ±0.66^{bc}	92.01 ±3.54^{b}	92.32 ±1.58^{a}	92.45 ±2.41^{a}
(-)-Epigallocatechin gallate	1200.23 ±10.56^{d}	1189.88 ±9.68^{e}	1201.00 ±15.15^{d}	1215.32 ±20.32^{cd}	1237.45 ±10.15^{c}	1260.55 ±9.58^{b}	1265.72 ±11.36^{b}	1367.91 ±15.47^{a}
Gallocatechin gallate isomer	8.01 ±0.08^{a}	7.96 ±0.12^{ab}	7.65 ±0.21^{b}	7.23 ±0.02^{c}	7.01 ±0.62^{d}	6.89 ±0.31^{e}	6.78 ±0.12^{ef}	6.65 ±0.09^{f}
B-type procyanidin dimer	150.23 ±2.65^{a}	149.34 ±3.15^{a}	143.67 ±1.09^{b}	143.21 ±2.44^{b}	143.01 ±1.06^{b}	135.87 ±2.58^{c}	130.81 ±2.65^{d}	127.15 ±1.25^{e}

(+)-Epicatechin gallate	55.36 ±1.3^{a}	53.81 ±0.08^{b}	50.23 ±1.06^{c}	45.82 ±1.21^{d}	40.45 ±0.89^{e}	40.02 ±1.41^{e}	36.92 ±1.06^{f}	30.46 ±0.33^{g}
SUM	4368.29 ±46.37^{c}	4274.39 ±31.87^{e}	4322.92 ±43.41^{d}	4348.13 ±56.19^{cd}	4377.18 ±28.46^{c}	4394.33 ±41.53^{b}	4396.6 ±41.38^{b}	4494.14 ±39.13^{a}
**Flavonols**								
Mirycetin glucoside	32.30 ±0.31^{b}	28.17 ±0.25^{e}	28.99 ±0.16^{de}	29.01 ±0.32^{de}	29.45 ±0.20^{d}	29.89 ±0.15^{cd}	31.16 ±0.24^{c}	35.17 ±0.09^{a}
Mirycetin galactoside	110.80 ±2.65^{a}	105.66 ±1.69^{b}	104.32 ±5.12^{b}	103.21 ±1.48^{b}	100.01 ±2.54^{c}	97.15 ±0.90^{cd}	75.56 ±3.16^{e}	61.12 ±4.85^{f}
Quercetin-3-O-glucosylrutinoside	61.15 ±1.03^{a}	60.94 ±0.98^{ab}	59.56 ±2.15^{b}	59.02 ±1.25^{bc}	58.43 ±0.68^{c}	57.99 ±0.78^{cd}	57.04 ±1.09^{d}	56.81 ±1.22^{de}
Kaempferol 3-O-p-coumaroylhexoside isomer	175.54 ±3.65^{a}	175.44 ±3.15^{ab}	173.90 ±0.29^{b}	170.04 ±0.01^{c}	168.33 ±1.52^{d}	164.23 ±2.48^{e}	161.27 ±1.09^{f}	158.12 ±2.55^{g}
Quercetin-3-O-glucosylrutinoside	31.15 ±0.28^{a}	30.22 ±0.32^{b}	30.01 ±0.90^{b}	29.13 ±0.15^{c}	28.98 ±0.10^{cd}	28.43 ±1.20^{d}	28.03 ±0.60^{de}	27.94 ±1.05^{e}
Quercetin 3-O-dirhamnosylhexoside	55.43 ±0.52^{a}	53.01 ±0.32^{b}	52.13 ±0.12^{c}	51.98 ±1.02^{c}	51.12 ±0.69^{cd}	50.78 ±0.58^{d}	50.35 ±0.84^{de}	50.12 ±0.36^{e}
Quercetin hexoside-deoxyhexoside	75.81 ±0.25^{a}	71.71 ±0.31^{b}	70.87 ±0.09^{c}	70.00 ±0.21^{d}	68.15 ±0.35^{e}	67.71 ±0.54^{ef}	62.99 ±1.03^{g}	61.54 ±0.90^{h}
Quercetin 3-O-glucoside	130.78 ±2.21^{d}	125.98 ±1.58^{e}	126.12 ±3.12^{e}	127.98 ±0.05^{de}	130.27 ±1.48^{d}	136.71 ±2.57^{c}	142.83 ±0.30^{b}	146.10 ±1.09^{a}
Quercetin 3-O-galactoside	50.67 ±1.55^{e}	55.30 ±3.12^{ab}	55.85 ±0.59^{a}	54.98 ±1.51^{b}	53.17 ±2.06^{c}	52.89 ±1.38^{cd}	52.56 ±0.65^{d}	52.13 ±1.07^{de}
Kaempferol 3-O-p-glucosylrutinoside isomer	20.25 ±1.03^{a}	18.76 ±0.58^{b}	17.88 ±0.57^{c}	17.03 ±0.98^{cd}	16.79 ±0.32^{d}	16.42 ±0.08^{de}	15.24 ±0.58^{f}	14.98 ±1.03^{fg}
Kaempferol 3-O-2'',6''-di-p-coumaroylglucoside	47.73 ±2.01^{a}	43.99 ±1.54^{bc}	44.12 ±0.99^{b}	43.20 ±0.00^{d}	42.90 ±1.35^{de}	41.80 ±2.22^{e}	40.90 ±1.07^{f}	39.99 ±1.14^{g}
Kaempferol 3-O-rutinoside isomer	37.90 ±0.99^{a}	36.16 ±1.32^{b}	35.87 ±1.05^{b}	33.82 ±2.05^{c}	32.27 ±0.30^{d}	31.97 ±0.54^{d}	30.35 ±0.02^{de}	29.82 ±1.33^{e}
Isorhamnetin 3-O-rutinoside	50.07 ±1.28^{a}	46.44 ±2.06^{b}	45.70 ±1.15^{c}	45.66 ±0.69^{d}	44.33 ±1.15^{e}	43.67 ±1.23^{f}	42.90 ±0.89^{g}	42.51 ±0.58^{h}
Kaempferol 3-O-glucoside	0.00 ±0.00^{a}	0.00 ±0.00^{a}	0.00 ±0.00^{a}	0.00 ±0.00^{a}	0.00 ±0.00^{a}	0.00 ±0.00^{a}	0.00 ±0.00^{a}	0.00 ±0.00^{a}
Kaempferol 3-O-galactoside	90.12 ±3.12^{a}	89.45 ±2.58^{ab}	88.38 ±0.28^{b}	87.93 ±0.17^{bc}	87.24 ±1.65^{c}	87.03 ±0.35^{cd}	86.49 ±1.23^{de}	85.46 ±0.32^{e}
Isorhamnetin derivtive	62.00 ±3.36^{a}	61.50 ±0.15^{ab}	61.21 ±1.25^{b}	60.67 ±2.58^{c}	60.36 ±1.06^{cd}	60.25 ±1.11^{d}	59.82 ±3.28^{de}	59.63 ±2.15^{e}
Quercetin 3-O-acylglycoside isomer	66.39 ±2.09^{a}	56.90 ±1.08^{c}	57.12 ±1.11^{b}	57.01 ±2.25^{b}	56.98 ±1.09^{bc}	56.85 ±2.35^{c}	56.34 ±2.53^{d}	56.21 ±1.74^{d}
Quercetin 3-O-p-coumaroyldirhamnosylhexoside isomer	69.05 ±1.85^{a}	67.15 ±1.26^{b}	66.79 ±0.99^{c}	65.75 ±0.03^{d}	65.23 ±1.54^{de}	64.88 ±1.36^{e}	64.05 ±1.17^{ef}	63.52 ±0.02^{f}
Kaempferol 3-O-p-coumaroylrhamnosyldihexoside isomer	28.20 ±0.23^{a}	24.34 ±1.25^{e}	27.39 ±0.09^{b}	27.06 ±1.33^{b}	26.76 ±1.54^{c}	26.64 ±1.06^{cd}	26.54 ±0.58^{d}	26.43 ±1.23^{de}
Isorhamnetin-acylated-hexoside	1.28 ±0.12^{a}	1.26 ±0.04^{b}	1.28 ±0.10^{a}	1.24 ±0.08^{c}	1.23 ±0.07^{c}	1.19 ±0.06^{d}	1.18 ±0.02^{de}	1.17 ±0.00^{e}
Isorhamnetin-acylated-hexoside	3.50 ±0.12^{a}	3.34 ±0.08^{b}	3.20 ±0.11^{c}	3.16 ±0.13^{cd}	3.00 ±0.06^{d}	2.98 ±0.04^{d}	2.92 ±0.02^{de}	2.80 ±0.08^{e}
SUM	1200.12 ±49.62^{a}	1155.72 ±40.98^{b}	1150.69 ±35.04^{b}	1137.88 ±28.22^{c}	1125.00 ±34.21^{d}	1119.46 ±36.34^{d}	1088.52 ±35.32^{e}	1071.57 ±39.49^{e}
**Purine Alkaloids**								
Theanine	1.00 ±0.01^{a}	0.96 ±0.02^{a}	0.85 ±0.01^{b}	0.75 ±0.02^{c}	0.80 ±0.01^{c}	0.83 ±0.02^{bc}	0.85 ±0.02^{b}	0.86 ±0.02^{b}
Theobromine	7.55 ±0.08^{d}	7.40 ±0.15^{e}	7.60 ±0.21^{cd}	6.63 ±0.16^{f}	7.69 ±0.24^{c}	7.72 ±0.16^{bc}	8.01 ±0.30^{b}	8.95 ±0.14^{a}
SUM	8.55 ±0.16^{c}	8.36 ±0.29^{d}	8.45 ±0.38^{d}	7.38 ±0.31^{e}	8.49 ±0.43^{cd}	8.55 ±0.31^{c}	8.86 ±0.55^{b}	9.81 ±0.28^{a}
**Theaflavins**								
Theaflavin isomer	5.09 ±0.06^{a}	4.81 ±0.02^{ab}	4.78 ±0.00^{b}	4.71 ±0.03^{bc}	4.65 ±0.04^{c}	4.60 ±0.05^{cd}	4.57 ±0.00^{d}	4.55 ±0.01^{d}
Theaflavin isomer	1.43 ±0.02^{a}	1.36 ±0.01^{b}	1.27 ±0.02^{c}	1.20 ±0.02^{d}	1.01 ±0.00^{e}	0.23 ±0.01^{f}	0.04 ±0.00^{g}	0.00 ±0.00^{h}
SUM	6.52 ±0.14^{a}	6.17 ±0.05^{b}	6.05 ±0.03^{b}	5.91 ±0.09^{c}	5.66 ±0.07^{d}	4.83 ±0.10^{e}	4.61 ±0.00^{f}	4.55 ±0.02^{g}
**Flavones**								
Apigenin C-hexoside-C-pentoside	25.42 ±0.54a	24.87 ±0.43b	25.25 ±0.28a	24.55 ±0.10bc	24.35 ±0.19c	24.08 ±0.14cd	23.99 ±0.23d	23.90 ±0.09d
TOTAL	5955.51 ±96.82^{a}	5787.31 ±73.63^{e}	5836.86 ±79.14^{d}	5845.76 ±84.91^{cd}	5861.52 ±63.36^{c}	5868.21 ±78.43^{c}	5834.82 ±77.47^{d}	5914.57 ±79.00^{b}

Means of three separate analyses ± standard deviation. Duncan's test reveals significant differences ($p < 0.05$) between values in the same rows with different letters (a-h).
